# Acute viral encephalitis impacts dense‐core amyloid plaque pathology and dysregulates myeloid responses to amyloid plaques

**DOI:** 10.1002/alz.71637

**Published:** 2026-06-26

**Authors:** Dominic Ibarra Javonillo, Susana Furman, Lucas Le, Kellie Fernandez, Jazmyn Mulford, Vidushi Singla, Roshni Jha, Kate Inman Tsourmas, Nellie E. Kwang, Kim N. Green, Thomas E. Lane

**Affiliations:** ^1^ Department of Neurobiology & Behavior University of California Irvine California USA; ^2^ Deparment of Molecular Biology & Biochemistry University of California Irvine California USA; ^3^ Center for Virus Research University of California Irvine California USA

**Keywords:** 5xFAD, Alzheimer's disease, amyloid‐beta neuropathology, coronavirus infection, myeloid cells, spatial transcriptomic imaging, viral encephalitis

## Abstract

**INTRODUCTION:**

Recent epidemiological datasets have associated viral encephalitis exposure (i.e., viral‐induced neuroinflammation) with increased risk of Alzheimer's disease (AD) and dementia, highlighting the need to uncover how it may impact AD neuropathology.

**METHODS:**

Aged 5xFAD and wild‐type (WT) mice were infected with the John Howard Mueller strain of murine hepatitis virus (JHMV), a neurotropic strain of murine coronavirus to comprehensively determine how coronavirus‐induced encephalitis may induce molecular and cellular changes that impact beta‐amyloid (Aβ) neuropathology.

**RESULTS:**

JHMV‐induced encephalitis at 12 days post‐infection resulted in minimal changes to overall Aβ protein, despite increased CD4^+^ and CD8^+^ T‐cell infiltration and *Lgals3/*MAC2‐expressing macrophages surrounding more compact Aβ plaques in the brain. Spatial transcriptomic imaging and pathway analysis of differentially expressed genes (DEGs) within myeloid cells demonstrate down‐regulated disease‐associated (DAM) pathways involving Aβ clearance, response to lipids, and macrophage activation within infected 5xFAD brains.

**CONCLUSIONS:**

JHMV encephalitis induces dysregulated gene expression and myeloid cell responses to Aβ plaque burden in 5xFAD mouse brains.

## Highlights

- Viral encephalitis has been identified as a risk factor for Alzheimer's disease.
- John Howard Mueller strain of murine hepatitis virus (JHMV) encephalitis in 5xFAD mice resulted in infiltration of immune cells and reduced dense‐core plaque pathology, despite minimal changes to overall beta‐amyloid (Aβ) protein.
- HMV encephalitis downregulated myeloid genes associated with Aβ clearance, response to lipids, and macrophage activation in 5xFAD brain.

## 1 BACKGROUND

Alzheimer's disease (AD) is the most common cause of dementia impacting almost 7 million people in the United States with cases projected to increase over 13 million by 2050.^1^, ^2^ As a progressive neurodegenerative disease, AD‐associated neuropathology include widespread aggregation of neuritic beta‐amyloid (Aβ) plaques and neurofibrillary tau tangles (NFTs), which are proposed to occur consecutively or work synergistically towards neuroinflammation, neuronal loss, and eventual cognitive decline.^3^, ^4^ Genome‐wide association studies (GWAS) have been a critical tool to determine polymorphic variants within alleles that increase genetic risk and identify physiological processes contributing to AD pathogenesis in late‐onset AD.^5^ Numerous large GWAS have repeatedly identified AD risk genes associated with immunity and specifically expressed in myeloid cells, implicating neuroimmune processes and neuroinflammation as a major factor of AD etiology. Myeloid cells within the central nervous system (CNS) are primarily comprised of two distinct populations: CNS‐resident microglia and monocyte‐derived macrophages. Many studies have demonstrated a close association between Aβ plaques and microglia, which are considered the principal immune cell within the CNS. Once activated, microglia undergo a morphological and functional change into rounder, amoeboid shapes with short, thick processes that extend out to enable migration towards the inflammatory stimulus, cytokine secretion, and phagocytosis.^6^, ^7^

Microbial infection has been broadly explored as a mechanism through which both genetic and environmental contributions interact together to influence AD risk and neuropathology. Pathogenic microbes that invade the CNS can cause significant neuroinflammation through CNS‐resident immune cells and infiltration of peripheral immune cells such as monocyte/macrophages and T cells. To illustrate the association of viral infection with several neurodegenerative diseases, Levine et al.^8^ mined medical record data from both the Finnish biobank (FinnGen) and UK Biobank. Their findings demonstrated several viral pathogens tightly associated with different neurological diseases, while the strongest association was between AD and viral encephalitis.^8^ Viral encephalitis is defined as viral‐mediated inflammation of the brain parenchyma. While the blood–brain barrier (BBB) prevents most viral pathogens from neuroinvasion, neurotropic viruses can circumvent these barriers to infect and replicate within the CNS.^9^ The resulting inflammation via indirect neuroimmune mechanisms may induce acute and chronic neurologic damage with potential consequences in the onset or severity of neurodegenerative disease.^10^, ^11^, ^12^ Therefore, it is unsurprising that majority of identified viral pathogens associated with neurodegenerative disease and dementia are neurotropic (i.e., herpes simplex virus [HSV], varicella zoster virus [VZV], Epstein–Barr virus, and flaviviruses) and systemic infections leading to viral encephalitis (i.e., human immunodeficiency virus (HIV) and severe acute respiratory syndrome‐coronavirus 2 (SARS‐CoV‐2).^13^, ^14^, ^15^ While the field's current understanding of the contributions of these viruses and their resulting inflammation on AD remains nuanced and complex, ongoing investigations are required to precisely identify direct and indirect viral mechanisms and inflammatory pathways impacting AD neuropathology.

The neurotropic John Howard Mueller (JHM) strain of murine hepatitis virus (JHMV, a member of the *Betacoronaviridae* genus) has previously been utilized to model molecular and cellular mechanisms of host defense and disease in response to viral encephalitis within the CNS.^16^, ^17^ Intracranial inoculation of susceptible mice with JHMV results in an acute encephalomyelitis characterized by wide‐spread replication of virus in glia with relative sparing of neurons. The resulting innate immune responses include expression of type I interferon, which limit the spread of virus early following infection. Ultimately, virus‐specific CD4^+^ and CD8^+^ T cells are attracted into the CNS in response to T‐cell chemoattractant chemokines and control viral replication via expression of anti‐viral cytokines including interfero‐gamma (IFN‐γ) and cytolytic activity.^18^, ^19^ However, sterile immunity is not achieved and virus will persist within white matter tracts, where immune‐mediated demyelination will occur due to chronic infiltration of macrophages and T cells.^20^, ^21^ Based upon evidence that neuroinflammation impacts AD‐associated neuropathology and that viral encephalitis is now recognized as a major risk factor associated with AD, this study utilizing JHMV infection of aged C57BL/6 and 5xFAD mice to determine the molecular and cellular mechanisms by which viral encephalitis impacts existing AD pathology and neuroinflammation.

**RESEARCH IN CONTEXT**

1. **Systematic review**: The authors reviewed literature using traditional sources (i.e. PubMed), in addition to meeting abstracts and presentations. Although John Howard Mueller strain of murine hepatitis virus (JHMV) encephalitis and its impact on Alzheimer's disease (AD) neuropathology has not been studied, several publications investigating neurological consequences of viral‐induced inflammation in the central nervous system have highlighted its impact on neuronal health, neuroinflammation, and cognitive function. These publications have been appropriately reviewed and cited.
2. **Interpretation**: This study demonstrates that JHMV encephalitis in 5xFAD mice induces the infiltration of peripheral immune cells, including T cells and monocyte/macrophages. While dense‐core beta‐amyloid (Aβ) plaques become more compact, JHMV encephalitis does not impact overall Aβ protein concentration. Furthermore, JHMV encephalitis induces significant transcriptional changes across several cell types in the 5xFAD brain. In myeloid cells, JHMV encephalitis down‐regulates expression of genes associated with Aβ clearance, lipid responses, and macrophage activation.
3. **Future directions**: This study highlights the transcriptional impact of JHMV encephalitis in the 5xFAD mouse model, in addition to consequential changes in cellular responses to existing Aβ pathology. Future experiments will be required to determine whether these molecular changes impact the progression of Aβ deposition, in addition to neurofibrillary tau development utilizing tauopathy mouse models. Identifying these molecular mechanisms contributing to viral‐induced gene expression changes in the aging AD brain will yield beneficial therapeutic targets to protect against neurological consequences associated with viral encephalitis.

## 2 METHODS

### 2.1 Mice

Male and female C57BL/6 WT and 5xFAD at 6‐ and 10‐months of age were used for these studies. Tg (APPSwFlLon, PSEN1*M146L*L286V) 6799Vas/ Mmjax RRID:MMRRC034848‐JAX, was obtained from the Mutant Mouse Resource and Research Center (MMRRC) at The Jackson Laboratory, a National Insitutes of Health (NIH) ‐funded strain repository, and was donated to the MMRRC by Robert Vassar, Ph.D., Northwestern University and have been previously described and characterized in detail.^22^, ^23^ In brief, the 5xFAD mouse expresses five familiar AD genes (APP Swedish, Florida, and London; PSEN1 M146L+L286V) and recapitulate progressive amyloid pathology as early as 3‐4 months of age, leading to chronic neuroinflammation, synaptic loss, and dystrophic neurites. All studies were conducted following the NIH, American Physiological Society, and Universiy of California at Irvine (UCI) Animal Care Guidelines. Any necessary veterinary care was provided by vivarium technicians, who check on mice daily for any changes in the health or well‐being of the mice. Consistent with the recommendation of the Panel of Euthanasia of the AVMA, mice with continued discomfort and distress will be euthanized by inhalant overdose of CO_2_, followed by secondary euthanasia via cervical dislocation. All animal experiments involving mice were approved by UC Irvine Institutional Animal Care and Use Committee and conducted in compliance with all relevant ethical regulations for animal testing and research. All experiments involving mice also comply with the Animal Research: Reporting of in vivo Experiments (ARRIVE) guidelines.

### 2.2 Viral infection

All infections were performed on mice under deep anesthesia through intraperitoneal (i.p.) injections of a mixture containing 85 mg/kg ketamine and 10 mg/kg xylazine. Subsequently, mice were infected intracranially (i.c.) with 500 plaque‐forming units (PFU) of JHMV in 30 µL of sterile Hank's balanced sterile solution (HBSS); control mice receive i.c. injections only containing HBSS. Infected and control mice were weighed and monitored daily using a well‐accepted scoring criteria to screen clinical disease symptoms such as significant weight loss, inactivity, lack of grooming, and other indicators of distress or discomfort.^24^, ^25^

### 2.3 RNA extraction

Brains were isolated at defined times 12 days post‐infection (p.i.) was also homogenized with RNA extraction, cDNA synthesis, and quantitative polymerase chain reaction (qPCR) for comparison of JHMV Membrane (M) mRNA levels performed as previously described.^25^ Briefly, mouse brain tissue was added to TRIzol and homogenized using the Bead Ruptor 12 (Omni International) and 1.4 mm ceramic beads (Omni International, 19‐627). RNA was extracted via RNAeasy Minikit (Qiagen, 74106) using the “Purification of Total RNA, Including Small RNAs, from Animal Tissues” protocol from the manufacturer and Buffer RW1 to substitute Buffer RWT.

### 2.4 cDNA synthesis and qPCR

cDNA was made from extracted RNA using previously described methods.^26^ Briefly, cDNA was synthesized using the “First Strand cDNA Synthesis” protocol by New England Biolabs, using AMV Reverse Transcriptase (New England Biolabs, M0277L), Random Hexamers (Invitrogen, N8080127), RNAse Inhibitor (New England Biolabs M0314L) and AMV Buffer (New England Biolabs B0277A). Primer sequences used were glyceraldehyde‐3‐phosphate dehydrogenase (GAPDH) (forward: AACTTTGGCATTGTGGAAGG; reverse: GGATGCAGGGATGATGTTCT), JHMV Matrix glycoprotein (forward: TCAACCCCGAAACAAACAACC; reverse: GGCTGTTAGTGTATGG TAATCCTCA). qPCR was performed using the Bio‐Rad iQ5 and iTaq TM Universal SYBR© Green Supermix (Bio‐Rad, Hercules, CA). Reactions were performed using 10 µL and the machine was set to run using the following parameters: 1 cycle (95°C for 3 minutes) followed by 40 cycles (95°C for 10 seconds, then 55°C for 30 seconds). Ct values for each sample were normalized to an internal control (GAPDH), yielding dCt values where lower dCt values indicated higher mRNA levels present while higher dCt values represented lower mRNA levels as more cycles of amplification was required to detect a signal across threshold.

### 2.5 Cell Isolation and Flow Cytometry

Flow cytometry was performed to identify inflammatory cells entering the CNS using established protocols.^25^, ^27^ In brief, single cell suspensions were generated from tissue samples by grinding with frosted microscope slides. Immune cells were enriched via a two‐step Percoll cushion (90% and 63%), and cells were collected at the interface of the two Percoll layers. Before staining with fluorescent antibodies, isolated cells were incubated with anti‐CD16/32 Fc block (BD Biosciences, San Jose, CA) at a 1:100 dilution. Immuno‐phenotyping of cells was performed using commercially available antibodies specific for the following cell surface markers: CD4 (Invitrogen, 11‐0042‐82), CD8a (Invitrogen, 17‐0081‐82), CD11b (Abcam, ab24874), and CD45 (BioLegend, 103114; 103130). Cells were simultaneously incubated with LIVE/DEAD Aqua Dead Cell Stain (Invitrogen, L34966). The following flow cytometric gating strategies were employed for inflammatory cells isolated from the CNS: macrophages/myeloid cells (CD45 hi CD11b^+^) and microglia (CD45 lo CD11b^+^); fluorescein isothiocyanate (FITC) ‐conjugated rat anti‐mouse CD4 and a phycoerythrin (PE) ‐conjugated tetramer specific for the CD4 immunodominant epitope present within the JHMV matrix (M) glycoprotein, spanning amino acids 133‐147 (M133‐147 tetramer), to determine total and virus‐specific CD4^+^ cells;^28^, ^29^ APC‐conjugated rat anti‐mouse CD8a and a PE‐conjugated tetramer specific for the CD8 immunodominant epitope present in the spike (S) glycoprotein, spanning amino acids 510‐518 (S510‐518), to identify total and virus‐specific CD8^+^ cells.^28^, ^29^ Data were collected using a Novocyte flow cytometer and analyzed with FlowJo software (Tree Star Inc.).

### 2.6 Histopathology

The 6‐month and 10‐month‐old 5xFAD hemizygous and wild‐type (WT) non‐transgenic mice were euthanized by inhalant overdose of Isoflurane at 7, 12, or 14‐days post‐infection before opening the thoracic cavity as a secondary method of euthanasia. Before transcardial perfusion with 1× phosphate buffered saline (PBS), blood plasma was collected from mice via cardiac puncture. In all experiments, brains were dissected, and the hemispheres were divided along the midline. One hemisphere of each brain was fixed in 4% paraformaldehyde (PFA) in PBS for 24 hours at 4°C for immunohistochemical and spatial single‐cell transcriptomics. The other hemisphere was either fresh‐frozen in dry ice for sample preparation to perform biochemical and protein analysis or placed in an enzyme digestion mix for cell isolation and staining to perform Flow cytometry. Additionally, spinal columns were isolated and stored in 4% PFA for 24–36 hours prior to analyzing spinal cord histopathology using Luxol Fast Blue staining protocols.

### 2.7 Immunofluorescence staining

After 24 hours, PFA‐fixed brain hemispheres were transferred into 30% sucrose in 1× PBS for cryoprotection stored in 4°C for 48 – 72 hours before they were embedded in optimum cutting temperature (OCT) compound (VWR, Radnor, PA, USA) and stored in ‐80°C. Sagittal brain sections were cut at 30 µm using a cryostat (Thermo 95 664 OEC70 Micron HM525) and collected while free‐floating in an anti‐freeze solution of 30% ethylene glycol and 30% glycerol in 1× PBS kept in ‐20°C. Selected brain sections were chosen for immunohistological analyses between 1.10 mm and 1.95 mm lateral to Bregma). One representative brain section from each mouse within the identical experimental cohort (i.e., same age, sex, genotype, and experimental condition) were subjected to simultaneous staining in the same container as described.^22^, ^30^, ^31^ Briefly, the selected free‐floating brain sections underwent several washes, at room temperature unless otherwise stated as follows: three 1× PBS washes for 5 minutes. For Amylo‐Glo staining, free‐floating brain sections were washed in 70% ethanol for 5 minutes followed by rinsing with deionized water for 2 minutes before immersed in Amylo‐Glo RTD Amyloid Plaque Staining Reagent (1:100 dilution in 0.9% saline solution; TR‐200‐AG; Biosensis, Thebarton, South Australia) for 10 minutes per manufacturer's instructions. Post‐incubation, the free‐floating sections were rinsed with 0.9% saline solution for 5 minutes before a brief wash in deionized water for 15 seconds. After Amylo‐Glo staining, the brain sections continued onto standardized indirect immunohistochemical procedures as previously described.^22^, ^30^, ^31^ Briefly, sections were immersed in blocking serum solution (5% normal goat serum with 0.2% Triton‐X in 1× PBS) for 1 hour before an overnight incubation at 4°C in primary antibodies diluted in blocking serum solution. A complete list of antibodies with their respective dilutions is provided in Table 1. The following day, the brain sections were incubated with secondary antibodies for 1 hour in the dark after three washes of 1× PBS. Before mounting the brain sections onto microscope slides for imaging, the stained brain sections were once more rinsed in three washes of 1× PBS.

**TABLE 1:** List of primary and secondary antibodies used in this study.

Primary antibody	Source	Catalog number	Dilution	Secondary antibody	Source	Catalog number	Dilution
IBA1	Wako	09‐19741	1:2000	Goat anti‐rabbit IgG, Alexa Fluor 488	ThermoFisher	A11008	1:200
MAC2	Cedarlane	CL8942AP	1:500	Goat anti‐rat IgG Alexa Fluor 594	ThermoFisher	ab150160	1:200
6e10 Aβ1‐16	BioLegend	8030001	1:2000	Goat anti‐mouse, Alexa Fluor 555	ThermoFisher	A21424	1:200
OC	Sigma Aldrich	2286	1:1000	Goat anti‐rabbit IgG, Alexa Fluor 488	ThermoFisher	A11008	1:200
CD4	Abcam	ab183685	1:500	Goat anti‐rabbit IgG, Alexa Fluor 488	ThermoFisher	A11008	1:200
CD8	Abcam	ab217344	1:500	Goat anti‐rabbit IgG, Alexa Fluor 488	ThermoFisher	A11008	1:200

### 2.8 Confocal microscopy and Imaris quantitative analysis

Brain sections were imaged with a Zeiss Axio Scan Z1 Slidescanner using a 10 × 0.45 NA Plan‐Apo objective. High‐resolution confocal fluorescence images were also obtained using a 20 × 0.75 NA objective on a Leica TVS SPE‐II confocal microscope. Two brain regions per mouse were selected for imaging: subiculum (SUB) and somatosensory cortex (SS CTX), unless otherwise stated and one field of view (FOV) per brain region was obtained for image analysis using Bitplane Imaris Software for quantification. Cell counting and volumetric measurements were acquired using the Spots and Surfaces modules through batch analysis of each imaged brain region.

### 2.9 Protein extraction, biochemical analysis via meso scale discovery assays

Sample preparation and quantification of Aβ followed established protocols.^30^, ^31^ Hippocampal and cortical regions of each mouse brain hemisphere were micro‐dissected and flash‐frozen. Samples were pulverized using a Bessman Tissue Pulverizer. For Aβ biochemical analysis, pulverized hippocampal tissue was homogenized in 150 µL of Tissue Protein Extraction Reagent (TPER; Life Technologies, Grand Island, NY). Cortical tissue was homogenized in 1000 µL/150 mg of TPER. The formulation of TPER contains 25 mM bicine and 150 mM sodium chloride (pH 7.6) to effectively solubilize proteins within brain tissue post‐homogenization. Protease (Roche) and phosphatase inhibitors (Sigma‐Aldrich) were added to the homogenized samples and centrifuged at 100,000 g for 1 hour at 4°C to generate TPER‐soluble fractions. To further create formic acid fractions, pellets from TPER‐soluble fractions were homogenized in 70% formic acid: either 75 µL or half of the added TPER volume for hippocampus or cortical tissue, respectively. Samples were once again centrifuged at 100,000 x *g* for 1 hour at 4°C. Proteins in this insoluble fraction were normalized to the respective brain region weight, while protein in the TPER‐soluble fractions was normalized to the protein concentration determined via Bradford Protein Assay. Formic acid neutralization buffer (1 M TRIS base, 0.5 M Na_2_HPO_4_, 10% NaN_3_) was used to adjust pH before running enzyme‐linked immunosorbent assay (ELISA) assays. Quantitative biochemical analysis of human Aβ soluble and insoluble fraction levels were obtained using the V‐PLEX Aβ Peptide Panel 1 (6E10) (K15200G‐1; Meso Scale Discovery, Rockville, MD). Quantitative biochemical analysis of neurofilament‐light chain (NfL) in plasma samples was performed using the R‐Plex Human Neurofilament L Assay (K1517XR‐2; Mesoscale Discovery).

### 2.10 Luxol fast blue staining

Preparation for spinal cord histology was performed using previously described protocols.^24^, ^26^ In brief, PFA‐fixed spinal columns were dissected to carefully extract the spinal cord from thoracic vertebrae 6‐10 and cryoprotected in 30% sucrose for three days at 4°C. Spinal cords were then cut in 1 mm transverse blocks and subsequently embedded in optimum cutting temperature (OCT) compound (VWR, Radnor, PA, USA) and frozen at ‐80°C. Spinal cord tissue was then coronally cryosectioned with a thickness of 8 micrometers (µm) and mounted on slides for Luxol fast blue (LFB) staining using established protocols. Sections were stained with hematoxylin and eosin (H&E) in combination with LFB. Between 5‐8 sections per mouse spinal cord were imaged using brightfield microscopy for analysis of demyelination. Areas of total white matter and demyelinated white matter were obtained using FIJI ImageJ Software, while demyelination was scored as a percentage of total white matter from the analyzed spinal cord sections as previously described.

### 2.11 Bulk RNA sequencing

RNA was extracted from 6‐month‐old 5xFAD mouse brains infected with 500 PFU JHMV at 7‐ and 14‐days p.i. as described above. Library preparation, RNA sequencing, and read mapping analysis were performed by Novogene Co. Gene expression values were normalized into Log2 FPKM (fragments per kilobase of transcript per million mapped reads). Heatmaps were created using Morpheus (Morpheus, https://software.broadinstitute.org/morpheus). Volcano plots were created using custom code in R. Differentially expressed genes (DEGs) were filtered as significant with the magnitude of Log2 (Fold change) greater than 0.5 (|Log2FC| > 0.05) and a false discovery rate (FDR) < 0.05. For each experimentally relevant comparison, gene ontology (GO) term enrichment analysis was performed on significant DEGs using enrichR (https://amp.pharm.mssm.edu/Enrichr/).

### 2.12 Spatial transcriptomic tissue preparation

PFA‐fixed brain hemispheres were embedded in OCT and stored frozen at ‐80°C. 24 hours prior to processing tissue for CosMx Spatial Molecular Imaging (SMI), sagittal brain sections were cut at 10 µm and mounted on VWR Superfrost Plus microscope slides (Avantor, 48311‐703). In each slide, six sagittal sections equally representing each experimental group were mounted and allowed to dry at room temperature for 15 minutes to promote tissue adherence and stored overnight at ‐80°C with desiccant. The following day, the brain tissue slides were processed (a total of 12 brain sections) in accordance with the Bruker Nanostring CosMx specifications. Unless otherwise noted, the following steps were performed at room temperature under a fume hood in a cleaned, RNAse free area. Slides were dried in an oven for 60 minutes at 60°C before briefly incubated in pre‐cooled 10% neutral‐buffered formalin for 15 minutes at 4°C and rinsed in three 1× PBS washes afterward. The slides were placed back into the oven at 60°C for 45–60 minutes to further improve tissue adherence. After three washes in 1× PBS for 5 minutes, the slides were incubated in 4% sodium dodecyl sulfate (SDS) for 2 minutes, then washed in three washes of 1× PBS before dehydrating the tissue in subsequent 50%, 70% and 100% ethanol washes for 5 minutes each. After allowing the slides to air dry flat for 10‐30 minutes, the slides were placed in a pre‐heated container of 1× CosMx Target Retrieval Solution (Nanostring) at 100°C for 5 minutes, maintained by a steamer set to 100°C. After the antigen retrieval step, the slides were cooled in DEPC‐treated water (ThermoFisher Scientific, AM9922) for 15 seconds and 100% ethanol for 3 minutes before 30 minutes of air drying. To permeabilize tissue, the sections were incubated in a digestion buffer (3 µg/mL Proteinase K in 1× PBS; Nanostring) for 30 minutes before a rinse in NBF Stop Buffer (0.1 M Tris‐Glycine Buffer, Cat# 15740) for 5 minutes, then rinsed in three 1× PBS washes for five minutes each. Meanwhile, a fiducial solution (Nanostring) was prepared to a 0.0015% dilution in 2× SSC‐T and applied on tissue for 5 minutes. From this point, the slides were shielded from light. After two 1× PBS washes, slides were post‐fixed in 10% NBF for five minutes and washed in two NBF Stop Buffer washes for five minutes each. Afterwards, the slides were left to incubate in an NHS‐acetate (100 mM; Cat#26777) solution for 15 minutes before being rinsed in 2× saline‐sodium citrate (SSC, Cat#AM9763) for 5 minutes. Finally, the tissue was left to incubate with probe mix containing segmentation markers for ribosomal RNA (rRNA), CosMx Mouse Neuroscience Panel, 1000‐plex, RNA with a custom RNA Add‐On set, and RNAse inhibitor in Buffer R (Nanostring). The slides were placed inside a staining tray and left inside oven set to 37°C for hybridization over 16‐18 hours overnight at which point, the hybridization probe mix was removed from the slides, and these were placed in two pre‐heated stringent washes (50% deionized formamide, CAT #AM9342) in 2× SSC at 37°C for 25 minutes each. After two washes in 2× SSC for 2 minutes, the tissue was incubated in 4′,6‐diamidino‐2‐phenylindole (DAPI) nuclear stain for 15 minutes and then washed in 1× PBS for 5 minutes. Meanwhile, a segmentation marker mix for glial fibrillary acidic protein (GFAP) and histones was prepared and left to incubate on the tissue for 60 minutes. After slides were rinsed in three 1× PBS washes for 5 minutes, the tissue was once again incubated in NHS‐Acetate before finally being rinsed in two 2× SSC washes for 5 minutes. Before loading into the CosMx Spatial Molecular Imaging instrument, adhesive flow cells (Nanostring) were placed in each slide to create a fluidic chamber for imaging according to manufacturer's instructions. Once loaded and processed in the machine according to the manufacturer's instructions for a two‐slide run, approximately 850 fields of view (FOVs, about 75 FOVs per brain section) was chosen to capture the following brain regions: cortical regions, corpus callosum, hippocampus, upper thalamus, and upper caudate striatal regions for each of the 12 brain sections. Imaging continued for approximately 6 days and the raw data was collected onto the Nanostring AtoMx online platform.

### 2.13 Spatial transcriptomic analysis

For spatial transcriptomic analysis, raw datasets were exported from Nanostring AtoMx as a Seurat object and processed with R 4.3.1 software as previously described.^31^, ^32^ Briefly, principal component analysis (PCA) and Uniform Manifold Approximation and Projection (UMAP) analysis was performed to reduce dataset dimensionality and visualize cell clustering at 1.0 resolution to yield 42 clusters. Clusters were manually annotated based on top transcript expression markers of known marker genes and its location in XY space. Furthermore, cell count and proportion plots were generated by plotting the number of cells in each cell type and scaling values relative to (1) normalized percentages per experimental group by obtaining the ratio of cell counts in each cell type‐group pair by the total number of cells and (2) dividing by the sum of the proportions across the cell type to account for differences in sample sizes. MAST was used on scaled expression data to enable differential gene expression analysis per cell type between group comparisons to compute the average difference as defined by the difference in log‐scaled average expression across the compared groups for each cell type. DEG scores were calculated by take the sum of the absolute log_2_ fold change values of all transcripts with statistically significant (p_adj_ < 0.05) differential gene expression patterns between two group comparisons for each cluster. To generate data visualization, we utilized ggplot2 3.4.4174.

### 2.14 Statistics

Sample sizes were calculated using power analyses based on pilot data (G*power parameters: power 0.95, Type I Error Rate 5%, Effect Size: 1.56) and on historical experience (i.e., mice lost due to acute infection or aging), while being powered to detect relevant sex differences. Animal groups are randomized at weaning, and age‐matched control cohorts will be generated alongside their respective experimental groups to maximize rigor and reproducibility. Data was analyzed using two‐tailed unpaired t‐tests for comparison between two groups or analysis of variance (ANOVA; one‐ or two‐way with mixed effects) for comparisons of up to four groups with appropriate post‐hoc tests (Holm‐Šidák's multiple comparisons) when necessary. For non‐parametric data, Kruskal–Wallis tests were used to compare group differences followed by Dunn's multiple comparisons test. Statistical significance is defined as *p* < 0.05 and statistical trends at *p* < 0.1. Read mapping analysis and statistics for bulk RNA sequencing data were performed by Novogene Co. Statistics for transcriptomic data (CosMx Spatial Molecular Imager) will be calculated using standard R packages, with a false discovery rate (FDR) < 0.05.

### 2.15 Availability of data and materials

Protocols, data, and results generated during the current study will be available via the AD Knowledge Portal (https://adknowledgeportal.synapse.org/). The AD Knowledge Portal is a platform for accessing data, analyses, and tools generated by the Accelerating Medicines Partnership (AMP‐AD) Target Discovery Program and other National Institute on Aging (NIA) ‐supported programs to enable open‐science practices and accelerate translational learning. The data, analyses, and tools are shared early in the research cycle without a publication embargo on secondary use. Data is available for general research use according to the following requirements for data access and data attribution (https://adknowledgeportal.org/DataAccess/Instructions).

## 3 RESULTS

### 3.1 5xFAD mice exhibit similar weight loss, mild clinical disease, and control of viral replication within the CNS following intracranial JHMV infection

**FIGURE 1:**
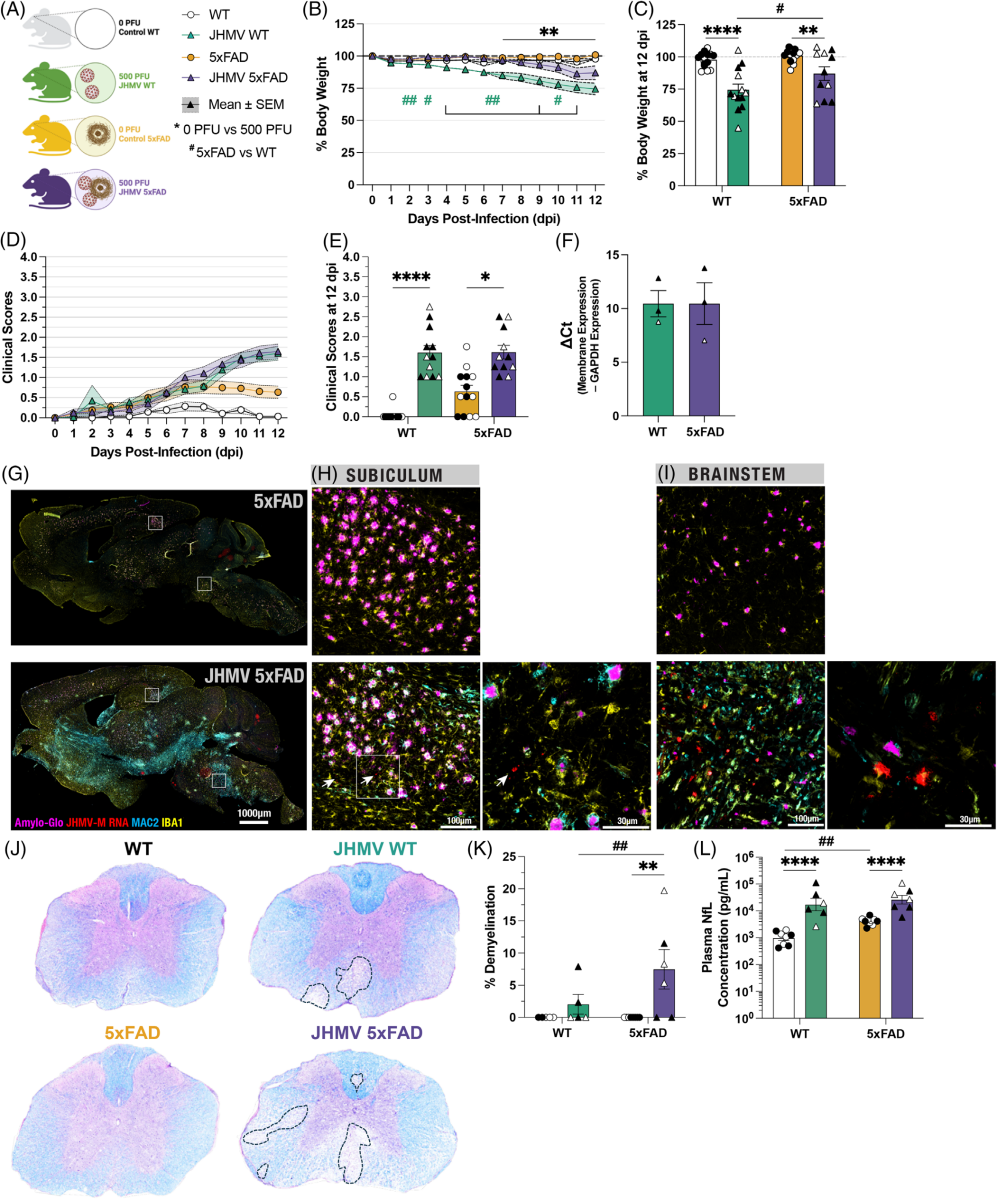
JHMV infection increases clinical disease severity and spinal cord demyelination by 12 days p.i. (A) Schematic of each experimental group. (B) Line chart depicting changes in initial body weights across each day post‐inf ection for uninfected WT (white), JHMV‐infected WT (green), uninfected 5xFAD controls (yellow), and JHMV‐infected 5xAFD mice (purple). (C) At 12 dpi, percent changes in body weight were compared across each experimental group. (D) Line chart depicting the progression of clinical disease severity and hindlimb paralysis across each day post‐infection. (E) At 12 dpi, clinical disease scores were compared across each experimental group. (F) qPCR with extracted RNA from brain homogenates demonstrate similar transcript levels of viral membrane protein RNA between JHMV‐infected WT and 5xFAD brains at 12 dpi. (G) Representative whole‐brain scanned images of sagittal brain sections from uninfected and JHMV‐infected 5xFAD brains co‐stained with Amylo‐Glo, MAC2, and IBA1 in addition to in situ hybridization targeting JHMV RNA. Representative 20× and 63× confocal images of subiculum (H) and brainstem regions (I) in brain sections of uninfected and JHMV‐infected 5xFAD mice. Fluorescent signals of viral RNA are indicated with white arrows. (J) Representative images of H&E/Luxol fast blue‐stained spinal cord sections across each experimental group. Dashed lines demarcate areas of demyelination. (K) The percentage of demyelination was compared across each experimental group. (L) Plasma concentration of NfL protein in blood plasma was compared across each experimental group. *n* = 7–10 per group from two independent experiments (*n* = 4‐5 mice per group per experiment). Data are presented as mean ± SEM. Unpaired *t*‐tests was used when appropriate and two‐way ANOVA followed‐by Holm–Sidak post‐hoc tests were performed to examine biological relevant interactions. Clinical disease scores were compared using Kruskal–Wallis tests followed by Dunn's multiple comparisons test. *p < 0.05, **p < 0.01, ***p < 0.001, ****p < 0.0001. Males are represented with closed symbols and females are represented with open symbols.

To determine whether familial AD mutations and its ongoing neuroinflammation impacted morbidity following JHMV infection of the CNS, we utilized 10‐month‐old 5xFAD and WT C57BL/6 mice to measure viral replication and onset of neurological disease during acute JHMV‐mediated encephalitis at 12 days post‐infection (p.i) (Figure 1A). By 12 days p.i., both JHMV‐infected WT mice and JHMV‐infected 5xFAD mice had significant reductions in body weight compared to uninfected controls (Figures 1B,C, *p* < 0.0001 and *p* < 0.01, respectively). When comparing the infected groups together, JHMV‐infected WT mice had greater body weight loss compared to JHMV‐infected 5xFAD mice (*p* < 0.05). When considering clinical disease severity assessed by hindlimb paralysis, both JHMV‐infected WT and JHMV‐infected 5xFAD mice displayed similar progression of motor impairment and clinical disease by 12 days p.i. (Figure 1D). By 12 days p.i., both JHMV‐infected WT and JHMV‐infected 5xFAD mice had significant progressive motor impairments compared to their uninfected controls (Figure 1E, *p* < 0.0001 and *p* < 0.05, respectively). Using qPCR to quantify levels of viral RNA in infected brains by 12 days p.i., no significant differences were observed between JHMV‐infected WT and JHMV‐infected 5xFAD, suggesting similar control over viral replication at 12 days p.i. (Figure 1F). This is corroborated in younger 6‐month‐old WT and 5xFAD mice, which also displayed similar levels of viral RNA via qPCR by 10‐14 days p.i. (Figure S1A).

### 3.2 JHMV viral RNA are present across similar brain regions in the CNS of infected WT and 5xFAD mice

Despite effective viral clearance, sterile immunity is not achieved and viral antigen and RNA will continue to persist within white matter of the CNS. Using antibodies against JHMV nucleocapsid protein, histological staining revealed presence of viral antigen within white matter regions (e.g., brainstem and peduncles) in addition to the subiculum hippocampal region within JHMV‐infected 5xFAD brains at 12 days p.i. (Figure S1B,C). Notably, viral antigen is not observed in somatosensory cortical regions. In addition to viral antigen, in situ hybridization (ISH) via RNAscope also reveals the widespread active replication of viral RNA throughout the WT brain at 7 days p.i. within specific regions where viral antigen is present, specifically the subiculum and brainstem (Figure S1D‐F). This is also corroborated in JHMV‐infected 5xFAD brains, which reveal a lingering presence of JHMV RNA in the subiculum region, but more so in the brainstem (Figure 1G‐I). Despite similar viral control efficacy, JHMV‐infected 5xFAD mice exhibited greater immune‐mediated demyelination in LFB‐stained spinal cord sections at 12 days p.i. compared to JHMV‐infected WT mice (Figure 1J‐K, *p* < 0.01). To assess levels of axonal damage resulting from JHMV infection, we measured concentration of plasma NfL. In line with previous studies, we found significant increases in plasma NfL protein concentrations in 5xFAD mice compared to WT mice (Figure 1L). Additionally, we found that JHMV infection further increased plasma NfL concentration in JHMV‐infected WT and 5xFAD compared to their respective, uninfected controls (Figure 1L).

### 3.3 Dense‐core Aβ plaque burden is reduced in JHMV‐infected 5xFAD mice in subiculum and somatosensory cortical regions

**FIGURE 2:**
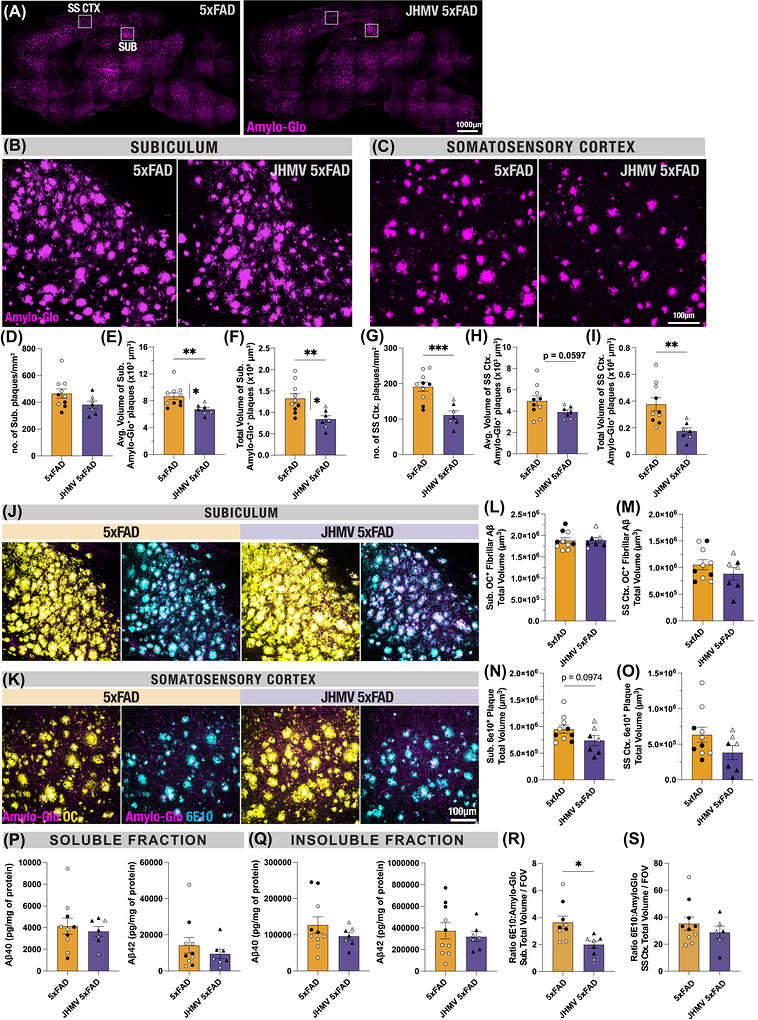
JHMV infection reduces dense‐core amyloid plaque pathology, but not overall Aβ burden. Aβ plaque burden was assessed using Amylo‐Glo staining for dense‐core plaques in 30 µm sagittal brain sections. (A) Representative whole‐brain images of 12dpi JHMV‐infected and control 5xFAD mouse brains stained with Amylo‐Glo. Representative 20× confocal images of subiculum (B) and somatosensory cortex (C) depicting Amylo‐Glo+, dense‐core Aβ plaques. Quantification of plaque densities (D, G), individual plaque volumes (E, H), and average plaque volumes (F, I) with the FOV of JHMV‐infected and control 5xFAD brain sections. Representative confocal images of brain sections costained with Amylo‐Glo, conformation‐specific OC antibody against amyloid fibrils, and 6E10 antibody against Aβ1‐16 residues in subiculum (J) and somatosensory cortex (K) of uninfected and JHMV‐infected 5xFAD mice. Quantification of OC fibrillar Aβ volumes around dense‐core plaques in subiculum (L) and somatosensory cortex (M). Quantification of 6e10+ diffuse plaque volumes in subiculum (N) and somatosensory cortex (O). Protein concentration of Aβ40 and Aβ42 within detergent‐soluble fractions (P) or insoluble fractions (Q) of frozen cortex homogenates. (R) Calculated ratio of 6E10+ diffuse Aβ total volume to Amylo‐Glo+ dense‐core plaque total volume in the subiculum. (S) Calculated ratio of 6E10^+^ diffuse Aβ total volume to Amylo‐Glo^+^ dense‐core plaque total volume in the somatosensory cortex. *n* = 7‐10 per group from two independent experiments (*n* = 4–5 mice per group per experiment). Data is presented as mean ± SEM. Unpaired t‐tests was used to examine statistically significant differences between groups. *p < 0.05, **p < 0.01, ***p < 0.001, ****p < 0.0001. Males are represented with closed symbols and females are represented with open symbols.

The significance of viral RNA present in the subiculum compared to the somatosensory cortex may suggest that areas of viral RNA persistence may influence Aβ plaque deposition in 5xFAD mice.^22^, ^33^ In JHMV‐infected 5xFAD brains, Amylo‐Glo staining for dense‐core Aβ plaques revealed significant reductions in Aβ plaque volume in the subiculum (Figure 2A–F, *p* < 0.01). However, in the somatosensory cortex where viral RNA was not observed, JHMV infection significantly reduced the number of dense‐core Aβ plaques with only a trending reduction in the average volume of Amylo‐Glo^+^ dense‐core plaques (Figure 2G–I).

To assess the role of JHMV infection on plaque composition, we utilized conformation‐specific OC antibody against fibrillar Aβ and 6E10 antibody against Aβ_1‐16_ peptides to visualize diffuse plaque structures associated with increased neurotoxicity.^34^ Interestingly, staining for OC^+^ fibrillar Aβ surrounding the denser plaques revealed no changes in either 5xFAD subiculum or somatosensory cortex regions following JHMV‐infection (Figure 2J, L–M). Staining for Aβ_1‐16_ residues with 6E10 demonstrated similar results, with only trending reductions in 6E10^+^ diffuse plaque volume within the subiculum following JHMV infection (Figure 2K, N,O). This is further corroborated by biochemical analysis demonstrating no differences in soluble and insoluble Aβ40 and Aβ42 protein concentration in cortex homogenates of JHMV‐infected and control 5xFAD brains via MULTI‐ARRAY assay (MSD) (Figure 2P‐K). Interestingly, we observed a reduced ratio of diffuse 6E10^+^ plaques to dense‐core Amylo‐Glo^+^ plaques in subiculum regions of JHMV‐infected 5xFAD mice compared to uninfected controls (Figure 2R,S), indicating an increased compaction of diffuse Aβ peptides into plaques following infection.^34^, ^35^ Overall, these results demonstrate JHMV infection and its resulting encephalitis may distinctly impact dense‐core plaques as opposed to overall Aβ burden in the 5xFAD mouse brain.

### 3.4 MAC2+ macrophages infiltrate into the CNS following JHMV infection and strongly correlate with brain regions demonstrating reduced Aβ plaque burden

**FIGURE 3:**
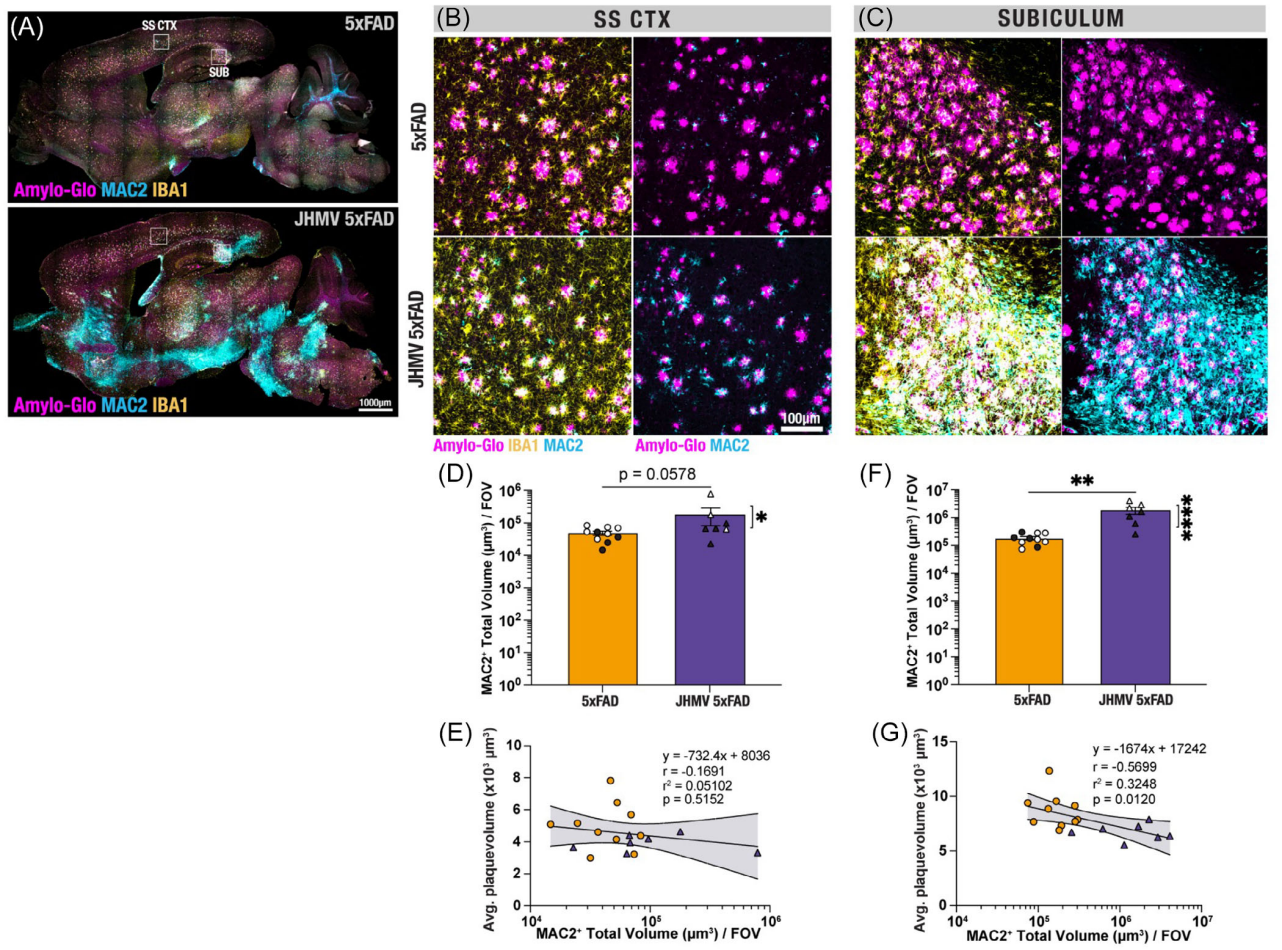
MAC2+ myeloid cells are associated with reduced dense‐core Aβ plaques in JHMV‐infected 5xFAD mice. Infiltration of peripheral myeloid cells (i.e., bone‐marrow‐derived monocytes and monocyte‐derived macrophages) was assessed using MAC2 antibody immunostaining of 30 µm sagittal brain sections. (A) Representative whole‐brain images of 12dpi JHMV‐infected and control 5xFAD mouse brains co‐stained with Amylo‐Glo, MAC2, and IBA1. Representative 20× confocal images of somatosensory cortex (B) and subiculum(C) depicting Amylo‐Glo+ dense‐core Aβ plaques surrounded by MAC2+ and IBA1+ macrophages. Quantification for total volume of MAC2+ cells in somatosensory cortex (D) or subiculum (F) of JHMV‐infected and control 5xFAD brain sections. Scatterplot and linear regression depicting no relationship in somatosensory cortex (E), yet a significant correlation between total volume of MAC2+ cells and average plaque volumes in subiculum (G) in 5xFAD mice. *n* = 7–10 per group from two independent experiments (*n* = 4–5 mice per group per experiment). Data is presented as mean ± SEM. Unpaired *t*‐tests was used to examine statistically significant differences between groups. Spearman correlation and linear regression was performed to measure the relationship between MAC2^+^ cell volume and Aβ plaque volume. **p < 0.01. Males are represented with closed symbols and females are represented with open symbols.

We have previously demonstrated that inflammatory *Lgals3*/MAC2‐expressing monocyte/macrophages migrate to areas of viral replication in response to chemokine expression using a combination of single‐cell RNA sequencing and histology.^36^ Immunostaining *Lgals3/*MAC2 in JHMV‐infected 5xFAD brains demonstrated high infiltration of peripheral monocyte/macrophages in the same brain regions where JHMV viral antigen is present, namely the subiculum, peduncles, and brainstem (Figure 3A). Representative confocal images of AD‐relevant brain regions like the somatosensory cortex and subiculum also illustrated localized interactions between MAC2^+^ and Aβ plaques (Figure 3B‐C). Moreover, quantifying the activation state MAC2^+^ cells via assessment of cell volume within AD‐relevant regions revealed increased volumes of MAC2^+^ cells within the somatosensory cortex (*p* < 0.0578) and a significant increase (*p* < 0.01) in volume of these cells in the subiculum (Figures 3D,F). Moreover, there was a significant (*p* < 0.05) correlation between enriched MAC2^+^ infiltration within the subiculum and associated with reduced Aβ plaque volumes (Figure 3G, *r* ^2 ^= 0.3248, *p* = 0.0120). These findings suggest that inflammatory monocyte/macrophages that are recruited into the CNS in response to JHMV replication during acute disease may contribute to morphology changes of dense‐core Aβ plaques.

### 3.5 CD4+ and CD8+ T cells infiltrate into the CNS following JHMV infection and tightly associate with MAC2+ interactions with Aβ plaques

**FIGURE 4:**
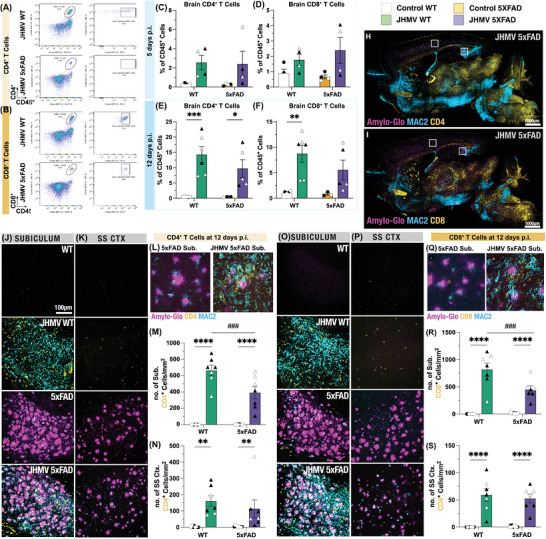
T cells infiltrate into the brains of JHMV‐infected WT and 5XFAD mice at 12 days p.i. Representative gating strategy to select CD45+ CD4+ T cells (A) and CD45+ CD8+ T cells (B) from JHMV WT and JHMV 5xFAD brains at 12 days p.i. following cell isolation, staining, and flow cytometry. Flow cytometric analysis of CD4+ T cell frequency (C) and CD8+ T cell frequency (D) from brain isolates at 5 dpi. Flow cytometric analysis of CD4+ T cell frequency (F) and CD8+ T cell frequency (F) from brain isolates at 12 dpi. Infiltration of CD4+ and CD8+ T cells into brain regions were assessed with brain sections co‐stained with Amylo‐Glo, MAC2, and either CD4 (H) or CD8 (I) antibody. Representative 20× confocal images of subiculum (J) and somatosensory cortex (K) depicting the presence of CD4+ T cells within brain sections from each experimental group. (L) Representative confocal images of control 5xFAD and JHMV 5xFAD subiculum. Quantification of CD4+ densities per FOV in subiculum (M) and somatosensory cortex (N) and within each experimental group. Representative 20× confocal images of subiculum (O) and somatosensory cortex (P) depicting the presence of CD8+ T cells within brain sections from each experimental group. (Q) Representative confocal images of control 5xFAD and JHMV 5xFAD subiculum. Quantification of CD8+ densities per FOV in subiculum (R) and somatosensory cortex (S) within each experimental group. Flow cytometry experiments includes *n* = 4–5 mice per group. Immunofluorescent staining includes *n* = 7 per group from two independent experiments (*n* = 3‐4 mice per group per experiment). Data is presented as mean ± SEM. Two‐way ANOVA followed‐by Holm–Sidak post‐hoc tests was performed to examine biological relevant interactions. *p < 0.05, **p < 0.01, ***p < 0.001, ****p < 0.0001. Males are represented with closed symbols and females are represented with open symbols.

To address the potential involvement of CNS inflammatory CD4^+^ and CD8^+^ T cells during acute JHMV infection in 5xFAD mice, flow cytometry was performed to quantify the frequency of CD45^+^ CD4^+^ and CD45^+^ CD8^+^ T cells at 5 and 12 days p.i. (Figure 4A,B). By 5 days p.i., there was moderate infiltration of both CD4^+^ and CD8^+^ T cells in both JHMV‐infected WT and 5xFAD brains, although no significance was detected (Figure 4C,D). However, both JHMV‐infected WT and 5xFAD brains exhibited significant levels of infiltrating CD4^+^ T cells at 12 days following JHMV infection compared to uninfected controls (Figure 4E, *p* < 0.001 and *p* < 0.05, respectively). Interestingly, JHMV‐infected 5xFAD brains showed a trending increase in infiltrating CD8^+^ T cells despite JHMV‐infected WT mice exhibiting a greater significant infiltration of CD8^+^ T cells compared to uninfected WT controls (Figure 4F, *p* < 0.01).

To visualize the infiltration of T cells into the brain parenchyma, we utilized fluorescent immunostaining of CD4^+^ and CD8^+^ T cells in tissue sections for confocal microscopy to also determine T cell populations in regions where reduced Aβ pathology was observed following viral encephalitis (Figures 4H,I). In parallel with the obtained flow cytometric data, JHMV‐infection mediated the infiltration of effector T cells, resulting in significant increases in the density of CD4^+^ T cells in both subiculum and somatosensory cortex regions of infected WT and 5xFAD brains at 12 days p.i. (Figure 4J–N). Furthermore, immunostaining for CD8^+^ T cells also reveals increased infiltration of CD8^+^ T cells in subiculum and somatosensory regions within infected WT and 5xFAD brains (Figure 4O–S). Interestingly, the infiltration of CD4^+^ and CD8^+^ T cells was significantly diminished in JHMV‐infected 5xFAD mice compared to JHMV‐infected WT mice (Figure 4 M,R).

### 3.6 Bulk RNA sequencing reveals upregulation of genes associated to host immunity and viral‐induced neuroinflammation in JHMV‐infected mice

To investigate transcriptional changes within 5xFAD brains following viral encephalitis, we isolated RNA from frozen hemi‐brain samples from each experimental group at days 7 and 14 p.i. and performed bulk sequencing analysis for dysregulated or DEGs for pathway analysis by gene ontology at each timepoint. Volcano plots were plotted to compare the average difference in gene expression (shown as Log_2_ fold change) in comparison with its respective control group. Genes were determined to be significantly dysregulated if the magnitude of average difference from baseline was greater than 0.5 with a false discovery rate/adjusted *p*‐value less than 0.05 (i.e. |Log_2_FC| > 0.5, FDR > 0.05). Significant DEGs were then plotted on volcano plots and heatmaps to illustrate its level of differential expression. JHMV‐induced encephalitis at both day 7 and 14 p.i. resulted in significantly down‐regulated DEGs in JHMV‐infected 5xFAD brains include *Sqle, Msmo1, Hmgcs1*, and *Gm9946*. Meanwhile, significantly DEGs in JHMV‐infected 5xFAD brains such as *Lyz2, Cd68, Gpnmb, Ctss, Ctsb, Ctsd, Lgals3, Il7r, Ly6c2*, and *Cxcl9* were significantly up‐regulated at day 7 p.i. (Figure S2A,J). Heatmap plots of the JHMV 5xFAD versus control 5xFAD comparison depict the magnitude of significant DEGs resulting from viral encephalitis (Figure S2B,K). Using GO, we conducted pathway analysis for down‐regulated pathways enriched in DEGs impacted by viral encephalitis in the 5xFAD brain and observed enriched pathways associated with cholesterol and steroid biosynthesis, neuronal transmission, axonogenesis, and lipid and lipoprotein metabolism. As expected, viral encephalitis in 5xFAD brains also up‐regulated pathways involved in immunity, such as the interleukin‐2 (IL‐2) signaling, cytokine signaling, T‐cell regulation of apoptosis, neutrophil activation, and lysosome functioning (Figure S2C,L). By day 14 p.i., several of these DEGs and enriched pathways impacted by viral encephalitis continue to be up‐regulated, in addition to *Spp1, Ccl5*, and *B2m* (Figures S2J–L).

To determine whether these changes were specifically associated with JHMV‐induced encephalitis, we have also performed bulk sequencing and DEG analysis on hemibrains from non‐transgenic WT mice infected with JHMV at 7 and 14 days p.i. (Figures S2D–F,2 M–O). Indeed, many significant DEGs and enriched pathways were also shared in JHMV‐infected WT mouse brains at 7 (Figures S2D‐F) and 14 days p.i. (Figures S3 M–O). Comparing gene expression differences between JHMV 5xFAD vs. JHMV WT mouse brains minimal differences in genes associated with immunity at day 7, yet several significant genes appeared up‐regulated in JHMV‐infected 5xFAD brains such as *Plin4, Hif3a, Chil3, Cdc25c, Fam107a, Gm29650*, and *Kdm5d*. Gene ontology analysis of significant DEGs in JHMV 5xFAD vs. JHMV WT brains reveal enriched down‐regulated pathways involved in BDNF signaling, while enriched up‐regulated pathways include hemostasis, platelet activation, IL‐6 regulation, and BBB transport (Figures 2G‐I,2P‐R).

### 3.7 Utilizing spatial transcriptomics to explore the impact of acute viral encephalitis on CNS cell types

**FIGURE 5:**
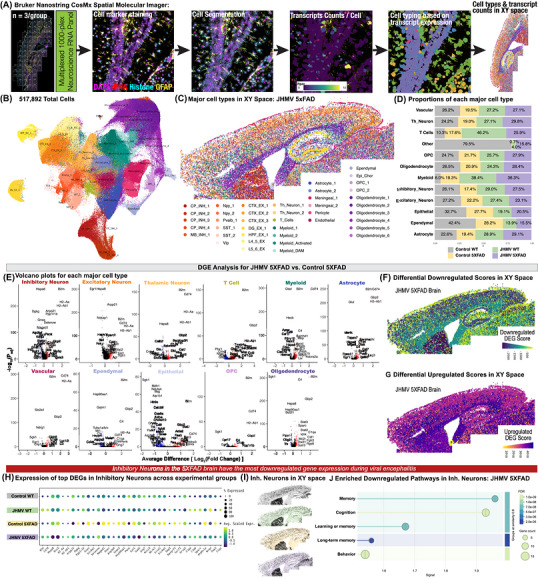
Spatial transcriptomic analysis of JHMV‐infected and control WT and 5xFAD mice. (A) Experimental workflow for targeted 1000‐plex single cell spatial transcriptomic imaging of 12 sagittal mouse brain sections (*n* = 3/group). FOVs were selected in dentate gyrus to represent the cell marker staining, segmentation, transcript counts, and cell typing based on transcript expression. DAPI, rRNA, Histone, and GFAP were used as cell markers. (B) UMAP of 517,892 cells across all brain samples were captured with a mean transcript count of 841 transcripts per cell. Unbiased cell clustering at 1.0 resolution identified 42 clusters, which were manually annotated with a combination of automated and manual approaches with reference to Allen Brain Atlas single‐cell RNA‐seq cell types, gene expression, and anatomic location in XY space. (C) 42 annotated clusters plotted in XY space with a representative JHMV‐infected 5xFAD brain. (D) Proportions of each major CNS cell type grouped by experimental group. (E) Volcano plots of DEGs within each major CNS cell type across JHMV‐infected 5xFAD versus control 5xFAD. (F) Differential downregulation (DD) and (G) differential upregulation (DU) scores for JHMV‐infected 5xFAD versus control 5xFAD in each cluster plotted in XY space using a representative JHMV‐infected 5xFAD brain. (H) Pseudo‐bulk expression of top DEGs in inhibitory neurons between JHMV‐infected 5xFAD versus control 5xFAD, grouped by experimental group. (I) Inhibitory neurons in XY space in representative brain sections from each experimental group. (J) Pathways enriched via GO of downregulated DEGs in JHMV‐infected 5xFAD versus control.

While these bulk sequencing methods have identified key biological pathways impacted by viral encephalitis in the 5xFAD brain, we lack the spatial and single‐cell resolution to determine which key cell types within the CNS are mediating changes associated with Aβ pathology. To determine the impact of acute viral encephalitis following JHMV infection on cells within the mouse brain we investigated transcriptional changes to myeloid cells and other CNS cell types using spatial transcriptomic imaging. This approach facilitates gene expression analysis with single‐cell resolution of CNS cell types, previously limited by technique‐induced expression changes associated with single‐cell and nucleus RNA‐seq.^31^ Furthermore, this technique retains gene expression of cells in distinct spatial regions of the brain, enabling in situ quantification and profiling of cell populations within a brain slice. We performed this experiment utilizing a high‐plex in situ analysis platform (Bruker Nanostring CosMx Spatial Molecular Imager) with a 950‐plex RNA mouse neuroscience panel and a 50‐plex Custom Add‐On RNA panel (Bruker Nanostring).^31^ Three sagittal hemibrain slices per group were processed capturing the cortex and hippocampus regions, resulting in a dataset comprising of 517,892 cells from 12 brain sections. The CosMx SMI platform performed cell segmentation using DAPI, histone, ribosomal RNA, and GFAP staining (Figure 5A, Figure S3A) and transcript counts per cell for each of the 1000 gene targets per cell were calculated with an average of 841 transcripts per cell for 517,892 cells (Figure 5B, Figures S3B,C).

Cell clustering was obtained using a community detection approach on a k‐nearest neighbor graph, followed by dimensionality reduction via UMAP. Clusters were manually annotated based on top gene expression, expression of canonical CNS cell markers, and its spatial XY location in the brain, identifying 42 annotated clusters comprised of 11 clusters of inhibitory neurons, seven clusters of excitatory neurons, two clusters of thalamic neurons, two clusters of astrocytes, four clusters of endothelial and vascular‐related cell types, four clusters of myeloid cells, two clusters of oligodendrocyte precursor cells (OPCs), six clusters of oligodendrocytes, one cluster of T cells, one cluster of ependymal cells, one cluster of epithelial cells, and one cluster designated “other” (Figure 5B,C, Figures S3D,E). Each of the 42 clusters were condensed into major clusters comprised of similar cell types (e.g., Astrocyte_1 and Astrocyte_2 were combined into a broader “Astrocyte” cluster) (Figure S3F). The proportions of each major cell type cluster were then plotted per experimental group to illustrate any changes associated with JHMV‐induced encephalitis, Aβ pathology, or both (Figure 5D, Figure S3F). Since cell clustering relies purely on gene expression levels and not spatial location, we verified the correct assignment of cell populations in neuroanatomical locations by assessing cell type proportions across each experimental group and by plotting cells in XY space (Figures S4A,B). For each cluster, we also determined cell counts across each cluster of cell types within the CNS (Figures S4C,D). Clusters with fewer than 500 cells (i.e., “Other”) were removed from downstream analysis. In concordance with histology and flow cytometry, we observed increased proportion of cells in myeloid and T cell clusters following JHMV encephalitis (Figures S4C,D).

To validate the impact of acute viral encephalitis on the transcriptional state of various cell types within the brain, we next conducted DGE analysis on JHMV‐infected WT and control, uninfected WT. Volcano plots depict several changes in gene expression within each CNS subtype and major cell type in the brains of these two groups (Figure S5,6A). To visualize the spatial distribution of all cell types with changes in gene expression, we generated a DEG score representing the magnitude of fold change between each comparison of two experimental groups. This was calculated using the sum of products between two variables: the average difference in gene expression between two groups and the negative log 10 p‐value for each gene.^31^ The calculated value provides each cluster with a value plotted in XY space, depicting the DEG score of all differentially down‐regulated (DD score) or up‐regulated genes (DU score) (Figures S6B,C). Notably, the myeloid cell cluster demonstrated the greatest DD score within JHMV‐infected WT brains, indicating the greatest down‐regulation of genes in this cell population. Visualizing the location of myeloid cells in XY space on a JHMV‐infected WT brain demonstrates that several myeloid cells exhibiting the most down‐regulation are found throughout cortical regions and the subiculum of the hippocampus, as well as the fornix (Figure S6B). Pathway analysis of down‐regulated DEGs within myeloid cells illustrate impacts on neuron projection morphogenesis, behavior, regulation of tissue and bone remodeling, and modulation of synaptic transmission (Figure S6D). Furthermore, myeloid cell populations significantly upregulate expression of several DEGs involved in gliogenesis, lymphocyte immunity, immune effector processes, and antigen‐presentation consistent with previous single‐cell sequencing analyses on JHMV infection of the CNS (Figure S6E).

In addition to myeloid cells also exhibiting high DU scores in JHMV WT brains, astrocytes and oligodendrocytes demonstrate the greatest magnitude of up‐regulated genes during acute viral encephalitis (Figure S6A). Indeed, visualizing DU scores of each CNS cell in XY space illustrates the highest scores along white matter areas such as the corpus callosum and fornix, but also distributed throughout the cortex and hippocampal formation (Figure S6C). Pathway analysis using GO of significantly up‐regulated genes within astrocytes and oligodendrocytes reveal biological processes associated with immune responses, immune effector processes, gliogenesis, and synaptic pruning during acute viral encephalitis (Figures S6F,G).

### 3.8 Spatial transcriptomic imaging reveals a shift in disease‐associated myeloid cells surrounding Aβ plaque pathology following acute JHMV‐induced encephalitis

To first determine the consequences of plaque deposition on cell types within the CNS, we performed DGE analysis on control 5xFAD and control WT brains. Volcano plots demonstrate the widespread transcriptional changes within each CNS subtype and major cell type impacted by plaque deposition (Figure S7,8A). In calculating the DEG score to compare the magnitude of fold change in DEGs within different CNS cell types, we found that myeloid cells demonstrated the greatest magnitude of down‐ and up‐regulated DEGs compared to other CNS cell types. Displaying the DEG score of each cell in XY space further depicts the distribution of dysregulated gene expression across myeloid cells in plaque‐laden areas, particularly the cortex and subiculum of 5xFAD mice (Figure S8B,C). In total, the presence of amyloid plaque deposition resulted in 190 significantly down‐regulated DEGs within myeloid cells (defined as p_adj _< 0.05 and absolute average difference > 0.3) between 5xFAD and WT brains. In line with previous sequencing studies, myeloid cells impacted by the presence of amyloid plaque pathology exhibited down‐regulated homeostatic genes (*P2ry12, Tmem119, Csf1r)*. Additionally, pathway analysis via gene ontology revealed down‐regulation in pathways involved in modulation of neurotransmission, cell morphogenesis, cell migration, development of neuron projections, and learning and memory (Figure S8D). Furthermore, myeloid cells exhibited 77 significantly up‐regulated DEGs between 5xFAD and WT brains, several of which are associated with disease‐associated microglia (DAM) signatures (*Cst7, Itgax, Spp1, Gpnmb, Ctss, Ctsz, Csf1, Lpl, etc.)* (Figure S8A). Meanwhile, additional up‐regulated DEGs within myeloid cells were associated with GO pathways involving regulation of tumor necrosis factor and IL‐6 production, gliogenesis and glial cell proliferation, and negative regulation of cell activation (Figure S8E).

We next performed DGE analysis to investigate how the acute viral encephalitis impacted CNS cell types surrounding Aβ plaque pathology. Non‐aggregate DGE analysis of JHMV‐infected 5xFAD vs. 5xFAD brains revealed dysregulated gene expression across the 42 cell clusters and major cell types (Figure S9 & Figure 5E). To visualize the spatial distribution of all cell types with changes in gene expression, we generated a DEG score representing the magnitude of fold change between each comparison of two experimental groups (Figures 5F,G). Visualizing down‐regulated DEG scores across brain sections demonstrates widespread genetic changes during acute JHMV‐induced encephalitis; however, inhibitory and excitatory neuron clusters exhibited the greatest DD score. Replotting the inhibitory neuron clusters in XY space validated the correct cell type clustering with spatial specificity. To visualize genetic changes in inhibitory neurons across the different experimental groups, we plotted the DEGs within this cluster as pseudo‐bulked expression values and in XY space (Figure 5H&I). Within inhibitory neurons in JHMV‐infected 5XFAD brains, several genes were downregulated involved in memory, cognition, and behavior such as *Cck, Cnr1, Vip, Sgk1*, and *Egr1* (Figure 5J).

Among all the cell types, myeloid cells expressed the greatest DU score between JHMV‐infected 5xFAD mice and uninfected 5xFAD controls, implicating a post‐acute encephalitis response that may interact with existing amyloid pathology. We observed an increased proportion of myeloid cells during viral encephalitis in 5xFAD brains, in concordance with increased infiltration following JHMV infection^26^ (Figure 5D). Using DGE analysis, we found that several genes associated with immune activation and antigen presentation are upregulated in the myeloid cell cluster (*CD74, H2‐Aa, H2‐Ab1;* Figure 5E). Interestingly, several DAM genes were downregulated within this myeloid population (*CD9, Trem2, Clec7a, Ctsz, Dtsd;* Figure 5E), as well as several homeostatic microglial genes (*Csf1r, P2ry12, Hexb, Cst3;* Figure 5E). To determine the biological pathways enriched in myeloid DEGs, we performed GO pathway analysis of dysregulated genes in the myeloid cluster. We found that JHMV‐infection induced several up‐regulated myeloid DEGs in 5xFAD brains associated with gliogenesis, glial cell development, glial cell differentiation, inflammatory responses, and myelination (Figure S10A). We also utilized protein network analysis to illustrate the protein‐protein interactions of closely associated up‐regulated myeloid DEGs (Figure S10B). Interestingly, GO pathway analysis of down‐regulated myeloid DEGs depicted enriched pathways involved in regulation of macrophage migration, host‐pathogen processes, ischemia responses, and neuronal death (Figure S10C,D).

**FIGURE 6:**
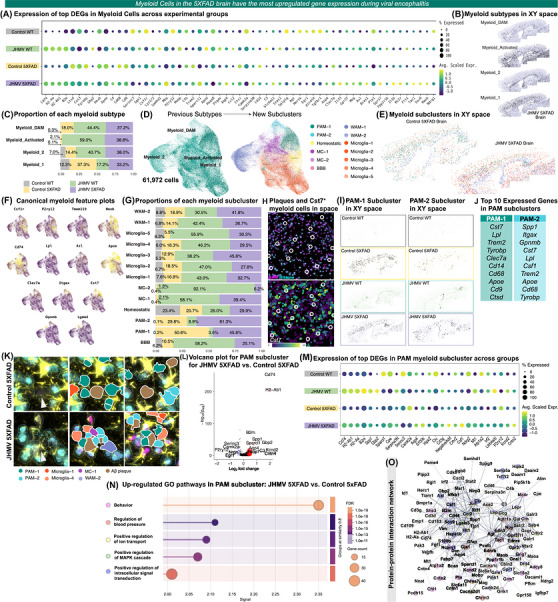
Sub‐clustering analysis of myeloid cells. (A) Expression of top DEGs in myeloid cells between JHMV‐infected 5xFAD versus control 5xFAD across each experimental group. (B) Myeloid cell subtypes in XY space on a representative section of a JHMV‐infected 5xFAD brain. (C) Proportion of each myeloid cell subtype split by each experimental group. (D) Myeloid cells were re‐clustered at 0.5 resolution yield 13 new myeloid subclusters. UMAP of 61,972 subsetted myeloid cells with original myeloid annotations (left) and new myeloid subcluster annotations (right). (E) Myeloid cell subclusters in XY space on a representative control 5xFAD and JHMV‐infected 5xFAD brain section. (F) Feature plots representing expression of several canonical myeloid cell markers on the new UMAP. (G) Proportion of the number of cells in each myeloid subcluster, grouped by experimental group. (H) Representative FOV of DAPI+ plaques and Histone‐expressing cells in the subiculum region of JHMV‐infected 5xFAD mouse (top). Cst7‐expressing cells in the same FOV (below). (I) PAM‐1 (left) and PAM‐2 (right) subclusters in XY space on representative sections from each experimental group. (J) List of the top 10 expressed genes in PAM‐1 (left) or PAM‐2 (right) subclusters. (K) Representative FOVs of subiculum in JHMV‐infected 5xFAD (top) and control 5xFAD (bottom) with arrows pointing to DAPI‐positive amyloid‐beta dense core plaques surrounded by myeloid subtypes. (L) Volcano plot for PAM subcluster in JHMV‐infected 5xFAD versus control 5xFAD. (M) Pseudo‐bulk sequencing analysis of top DEGs for JHMV‐infected 5xFAD and control 5xFAD within PAM myeloid subcluster. (N) Pathway analysis by Gene Ontology of enriched DEGs in the PAM myeloid subcluster for JHMV‐infected 5xFAD versus control 5xFAD comparison. (O) Protein–protein interactions (PPI) network of all significantly up‐regulated DEGs within PAM myeloid subcluster in JHMV‐infected 5xFAD versus control 5xFAD. Nodes are colored according to enriched pathway analysis. Connection line thickness between nodes indicates the degree of confidence in the prediction of the PPI.

Pseudo‐bulk analysis of these top DEGs between JHMV‐infected 5xFAD and 5xFAD brains was performed, demonstrating the downregulation of several DAM genes following acute viral encephalitis in 5xFAD myeloid cells despite DAM upregulation relative to control WT myeloid cells (Figure 6A). Cell clustering of all sampled cells produced four subtypes within the myeloid cluster: DAM, Activated, Myeloid 1, and Myeloid 2. Plotting these clusters in XY space reveals that the DAM and Myeloid 1 subtype to be distributed in plaque‐laden areas (i.e., the subiculum of the hippocampus and throughout the cortex; Figure 6B, Figures S11A,D). Meanwhile, the Activated Myeloid cluster appears to be pre‐dominantly distributed throughout the brain in regions associated with viral replication, whereas the Myeloid 2 population is primarily localized to white‐matter (i.e. the corpus callosum; Figure 6B, Figures S11B,C). Concordant with our histology and flow cytometry analysis, we found an increased proportion of cells within the DAM and Activated Myeloid subtype following acute viral encephalitis in both 5xFAD and WT brains (Figure 6C).

To further investigate the myeloid population, we captured all 61,972 myeloid cells across the four experimental groups and re‐clustered them into 13 new subclusters (Figure 6D). Once again, we performed manual cell annotation using top gene expression markers and their spatial location in the brains across each experimental condition to approximate an appropriate cluster annotation (Figure 6E, Figure S11E). For example, clusters highly expressing MHCII relevant markers *H2‐Ab1, H2‐Aa, and CD74* were highly enriched within JHMV‐infected WT and JHMV‐infected 5xFAD brains, suggesting that these clusters may present monocyte‐derived cells (MC) infiltrating into the CNS to control viral replication (Figure S11E‐I). Similarly, several clusters were found within brain regions enriched in white matter such as the corpus callosum and exhibited high expression of white‐matter associated genes such as *Mbp, Mobp, Mog*, and *Plp1*, suggesting that these captured cells represented white‐matter associated microglia (WAMs) and were tightly associated with oligodendrocytes Figure S11E). One cluster was equally represented across all experimental groups and displayed high expression of microglial genes like the homeostatic marker *P2ry12* and microglia‐specific marker *Siglech*, therefore we approximated that these likely represented homeostatic microglia (Figure S11E). Interestingly, we also observed several myeloid subclusters associated gene markers of different CNS cell types such as astrocyte markers *Gjal* and *Slca3* and neuronal markers *Camk2n1, Gria2, Syp, Pvalb*, etc. that were enriched across all experimental groups (Figure S11E‐I). Therefore, we labeled these clusters under the broader microglia annotation.

Among the sub‐clustered myeloid cells, we identified two subclusters predominantly found with 5xFAD brains, suggesting these clusters represented plaque‐associated microglia (PAM) subtype (PAM‐1 and PAM‐2). Visualization of cellular expression of canonical DAM markers *Lpl, Axl, Clec7a, Itgax, and Cst7* corroborates the identification of these PAM subclusters (Figure 6F). To demonstrate the expression of *Gpnmb* and *Lgals3* were predominantly within monocyte‐derived subclusters, we also visualized cells expressing these markers within the sub‐clustered myeloid UMAP (Figure 6F). In parallel to previous findings utilizing *Lgals3*/MAC2 as a potential marker for peripheral monocyte/macrophages,^36^ we observed *Lgals3* expression predominantly in monocyte‐derived cell clusters (Figure 6F). Cell proportions were calculated for each myeloid sub‐cluster across the four experimental groups, revealing a higher proportion of both PAM sub‐clusters are represented in 5xFAD brains (Figure 6G, Figure S11F‐I). While the PAM‐1 sub‐cluster is equally represented in both JHMV‐infected and control 5xFAD brains, we observed a greater proportion of cells in the PAM‐2 sub‐cluster in 5xFAD brains during viral encephalitis (Figure 6G, Figure S9I). In the cell segmentation imaging, we identified several DAPI‐positive “cells” that were only present in 5xFAD brains, anucleate, and lacked expression of histone markers, suggesting these to be DAPI‐positive Aβ plaques surrounded by *Cst7*‐expressing myeloid cells (Figure 6H).

Plotting these PAM subclusters in XY space demonstrates that both PAM‐1 and PAM‐2 subclusters are distributed throughout the 5xFAD brain in plaque‐laden areas, representing plaque‐associated myeloid cells (Figure 6I). Furthermore, both PAM‐1 and PAM‐2 sub‐clusters are characterized by high expression of canonical DAM markers (*Cst7, Lpl, Trem2, Apoe)* and phagocytosis (*Tyrobp, CD68)*, likely attributed to plaque‐associated microglia. However, cells within the PAM‐1 sub‐cluster differs in its high expression of *Clec7a, Cd9, and Cd14* while the PAM‐2 sub‐cluster exhibits a high expression of *Itgax, Csf1, Spp1, and Gpnmb* (Figure 6J). To determine the association amyloid pathology and cells with each PAM sub‐cluster, we examined the proximity of PAM‐1 and PAM‐2 cells surrounding Aβ plaques that stained positive DAPI but lacked histone markers (Figure 6K). We observed several PAM‐1 cells surrounding Aβ plaques in control 5xFAD mice, corroborating that PAM‐1 likely represent plaque‐associated microglia. Meanwhile, we confirmed a greater proportion of PAM‐2 cells surrounding Aβ plaques in JHMV‐infected 5xFAD brains. Furthermore, these PAM‐2 cells appear to be in direct contact with dense‐core Aβ plaque pathology following encephalitis, unlike PAM‐1 cells in control 5xFAD brains (Figure 6K). This data appears in line with our histological data, suggesting that cells within the PAM‐2 sub‐cluster could be responsible for the higher association of inflammatory MAC2^+^ myeloid cells surrounding Aβ plaques in response to JHMV infection.

To investigate the transcriptional changes that may occur in cells within the PAM sub‐cluster, we performed pseudo‐bulk analysis of DEGs across all the experimental groups (Figures 6L,M, Figure S12A). Relative to control 5xFAD brains, cells in the PAM sub‐cluster had increased expression of genes associated with immune activation and antigen‐presentation *(CD74, H2‐Ab1, H2‐Aa, B2m)* and IFN‐γ–induced signaling *(Gbp2, Irf2)* (Figures 6L,M). Gene ontology pathway analysis of these up‐regulated DEGs in PAMs depict effects on behavior, regulation of blood pressure, ion transport, regulation of MAPK cascade, and regulation of intracellular signal transduction (Figure 6N). To visualize the protein‐protein interactions and pathways associated with significant DEGs in PAM cells following viral encephalitis, we visualized interacting networks of related proteins expressed by up‐regulated DEGs (Figure 6O). In corroboration with our histological staining of *Lgals3/*MAC2 expression, we also found *Lgals3* among the up‐regulated DEGs within the PAM cluster in our spatial transcriptomic analysis, bolstering these transcriptional sequencing approaches with validation at the protein level through histology. Furthermore, performing DGE analysis on each PAM sub‐cluster reveals a differential impact of JHMV infection on PAM‐1 and PAM‐2 sub‐clusters in the 5xFAD brain (Figures S12B&C). In the PAM‐1 subcluster, pathway analysis illustrates enriched pathways for up‐regulated DEGs involved in regulation of MAPK cascade, gliogenesis, blood pressure, and secretion (Figure 12D). Down‐regulated genes within PAM‐2 cells enrich GO pathways involved in regulation of macrophage fusion, microglial cell activation, and responses to lipoprotein particles, while up‐regulated genes are associated with antigen presentation via MHC Class II, T‐cell activation, and regulation of adaptive immunity (Figure S12E).

Corroborating previous studies detailing the infiltration of monocyte‐derived macrophages during JHMV‐induced encephalitis,^26^ we have also observed high proportions of monocyte‐derived cells (MC) within JHMV‐infected WT and JHMV‐infected 5xFAD brains in our spatial transcriptomic myeloid dataset (Figure 6G, Figure S12F). Plotting the MC subclusters in XY space illustrates a significant spread of MC‐1 throughout the brains of JHMV‐infected mice, yet MC‐2 appears more specific to JHMV‐infected WT mice (Figure S12F‐G). Furthermore, *Lgals3* was identified to be highly expressed within the MC‐2 cluster (Figure S12G). When comparing transcriptional differences of monocyte‐derived cells within the MC subcluster between JHMV‐infected brains vs. Control 5xFAD brains, we find that JHMV infection results in decreased expression of *Lyz1, Mrc1, Tyrobp, Clec7a, Cd44*, and *Ctsd* (Figure S12H,I). Utilizing pseudo‐bulk sequencing analysis, we have also identified dysregulated DEGs among other myeloid cell types: WAM, Homeostatic myeloid cells, BBB‐associated myeloid cells, and microglia (Figure S13).

### 3.9 Comparison of myeloid differential gene expression effects of Aβ pathology and JHMV‐induced encephalitis

**FIGURE 7:**
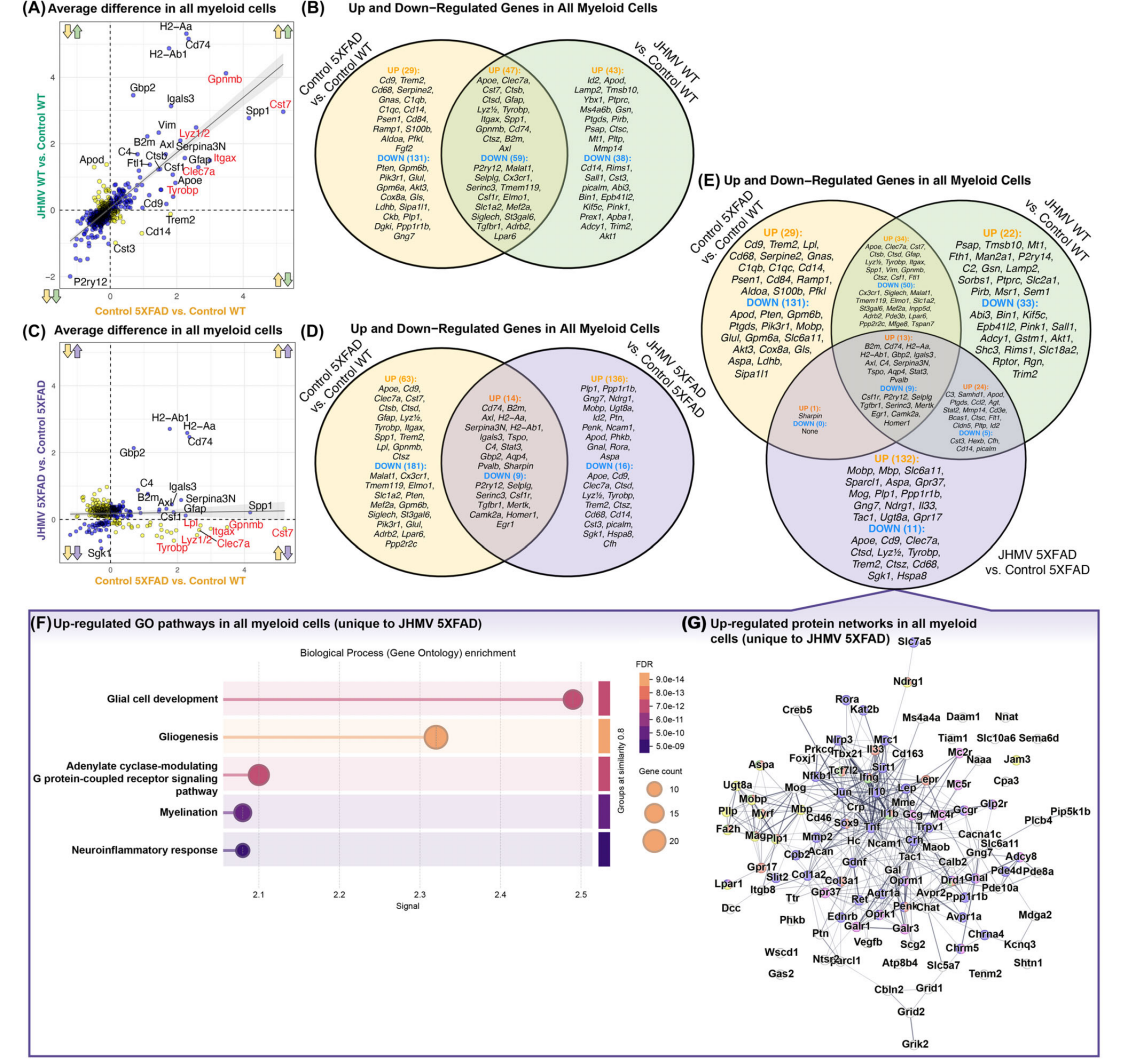
. Myeloid cells have differential responses to viral encephalitis, amyloid pathology, or both inflammatory stimuli concurrently. (A) Scatterplot of the average difference of all myeloid cells for all significant genes (padj < 0.05) between control 5xFAD versus control WT (*x*‐axis, only amyloid pathology) and JHMV WT versus control WT (*y*‐axis, only viral encephalitis) comparisons. Arrows indicate direction of dysregulation for each comparison. Directly correlated genes (blue) occur in the same direction for both comparisons (i.e., both up‐ or both down‐regulated), while inversely correlated genes (orange) occur in opposite directions for each comparison. Linear regression line demonstrates the relationship between the two comparisons. (B) Venn diagram depicting the significant up‐ and down‐regulated genes unique to the control 5xFAD versus control WT (left, yellow) and JHMV WT versus control WT (right, green), while demonstrating up‐ and down‐regulated genes commonly shared across the two comparisons (middle). (C) Scatterplot of the average difference of all myeloid cells for all significant genes between control 5xFAD versus control WT, now with JHMV 5xFAD versus control 5xFAD (*y*‐axis, both amyloid and viral encephalitis). Red text demonstrates genes up‐regulated in the same direction in the first scatterplot, but down‐regulated in the JHMV 5xFAD versus control 5xFAD comparison. (D) Venn diagram depicting the significant up‐ and down‐regulated genes unique to control 5xFAD versus control WT and JHMV 5xFAD versus control 5xFAD (right, purple), while demonstrating up‐ and down‐regulated genes commonly shared between the two comparisons. (E) Three‐way Venn diagram of all significantly up‐ and down‐regulated genes between the three comparisons: amyloid pathology (yellow), viral encephalitis WT (green), both amyloid and viral encephalitis (purple). Genes are considered significantly correlated if the log2 fold change magnitude is greater than 0.3. (F) GO pathways enriched by all significantly up‐regulated DEGs unique to JHMV 5xFAD versus control 5xFAD. (G) PPI of the significantly up‐regulated DEGs unique to the JHMV 5xFAD versus control 5xFAD. Nodes are colored according to enriched pathway analysis. Connection line thickness between nodes indicates the degree of confidence in the prediction of the PPI.

We have demonstrated that acute viral encephalitis through JHMV infection shifts the transcriptional signature, particularly in disease‐associated myeloid cells surrounding Aβ plaques. To investigate whether dysregulated responses in myeloid cells is a consequence of encephalitis, the presence of Aβ pathology, or unique to the presence of both inflammatory stimuli, we compared the average differences in gene expression within all myeloid cells in our spatial transcriptomic dataset. We plotted all DEGs between control 5xFAD versus control WT (response to amyloid pathology) and JHMV WT versus control WT (response to viral encephalitis) using the average difference of each gene among the two comparisons (Figure 7A). Many gene expression changes were shared and correlated between the two inflammatory stimuli. In response to either amyloid or JHMV encephalitis, myeloid cells shared down‐regulation of 59 genes with similar homeostatic markers (e.g., *P2ry12, Cx3cr1, Tmem119, Csf1r, etc*.), while sharing up‐regulation in 47 genes related to DAM signatures (*e.g., Apoe, Clec7a, Cst7, Ctsb, Itgax, Axl, etc.)*. Notably, several genes were more upregulated in response to one stimulus such as *H2‐Aa, Cd74, H2‐Ab1, Gpnmb, Lgals3*, which demonstrated greater upregulation in response to JHMV infection (Figure 7B). Meanwhile, there were several genes that were uniquely differentially expressed in one inflammatory stimulus over the other. For example, *Cd9, Trem2, Cd68, C1qb, C1qc* were upregulated within myeloid cells in response to amyloid pathology compared to JHMV infection. Conversely, *Cd14, Cst3, Picalm, Abi3, Bin1, Kif5c* were more downregulated in response to viral encephalitis compared to amyloid pathology (Figure 7B). Therefore, while the activation response to viral encephalitis and amyloid pathology similarly upregulated DAM‐related, inflammation genes, there were nuanced differences in related groups of genes when considering the magnitude of their inflammatory response.

To determine the effect of the concurrence of both inflammatory stimuli (e.g., amyloid pathology and viral encephalitis) on the gene expression within myeloid cells, we plotted all DEGs between control 5xFAD versus control WT and JHMV 5xFAD vs. control 5xFAD (Figure 7C). Once again, many differential gene expression changes were shared in the context of only amyloid pathology (Control 5xFAD) or *both* amyloid and viral encephalitis. Myeloid cells shared 14 up‐regulated genes involving MHC class II presentation, adaptive immunity activation, and other inflammatory mechanisms like *Cd74, B2m, H2‐Aa, H2‐Ab1, Stat3, Gbp2* (Figure 7D). Additionally, 9 homeostatic microglia genes were similarly downregulated in both conditions, such as *P2ry12 and Csf1r*.

Notably, to determine which set of DEGs were unique to the interaction of both amyloid pathology and viral encephalitis, we compared DEGs from all myeloid cells across three comparisons: Control 5XFAD versus control WT (only amyloid pathology), JHMV WT versus control WT (only JHMV infection), or JHMV 5XFAD versus control 5XFAD (both amyloid pathology and JHMV infection). We have identified 143 genes that uniquely become differentially expressed under the occurrence of both amyloid pathology and JHMV infection. Several DAM genes are among the 11 uniquely downregulated in the interaction of both inflammatory stimuli in JHMV‐infected 5xFAD brains: *Apoe, Cd9, Clec7a, Ctsd, Lyz1/2, Tyrobp, Trem2, Ctsz, and Cd68*. Notably, these same set of DAM genes were observed to be upregulated in the presence of both amyloid pathology and viral encephalitis, independently (Figure 7F). Additionally, 132 genes were observed to be uniquely upregulated in the interaction of both inflammatory stimuli (Figure 7F). Pathway analysis of these up‐regulated genes in JHMV 5XFAD brains reveal impacts on glial cell development, gliogenesis, adenylate cyclase‐modulating G protein‐coupled receptor signaling pathways, myelination, and neuroinflammatory responses (Figure 7G). Additionally, mapping out protein‐protein interactions of significantly up‐regulated DEGs unique to JHMV 5xFAD myeloid cells demonstrate closely linked genes that transcribe proteins associated with interactions or similar cellular mechanisms.

## 4 DISCUSSION

Emerging evidence from both human datasets, *post mortem* analysis, and in pre‐clinical AD models suggest that microbial infection and the resulting inflammation responses may interact with AD pathogenesis, serving as a major environmental risk factor for AD.^9^, ^10^, ^37^, ^38^, ^39^, ^40^ Importantly, viral exposure has recently been associated with increased risk of numerous neurodegenerative diseases, with the strongest association occurring between viral encephalitis and AD.^8^ There is a critical need to understand post‐infectious neuroimmune mechanisms and how they may interact with Aβ plaque deposition and modulate the onset and severity of AD pathology.

We demonstrate that acute JHMV infection in 5xFAD mice results in recruitment of inflammatory monocyte/macrophages and T cells within regions where viral RNA/antigen persists. In the subiculum and somatosensory cortex, we observed compaction of dense‐core Aβ plaques and reduced plaque numbers associated with MAC2^+^ macrophages, respectively. Furthermore, we utilized spatial transcriptomic imaging to investigate the transcriptional impact of viral encephalitis with single‐cell resolution and determine dysregulated gene expression within specific cell types surrounding Aβ pathology in the 5xFAD brain. While several cell types exhibited DEGs following viral encephalitis, we show that myeloid cells exhibit a down‐regulated DAM response in the brain of JHMV‐infected 5xFAD mice, particularly in cells those surrounding amyloid pathology. These findings reveal that viral encephalitis and its resulting inflammatory mechanisms impacts the expression of key genes within myeloid cells responding to Aβ plaque pathology, which has implications to the role of viral encephalitis as a risk factor for AD.

During neurodegenerative diseases like AD, microglia transition from homeostatic states into activated inflammatory states, which correlate with cognitive decline and neurodegeneration.^41^, ^42^ In AD, the prominent transcriptional signature and functional state within activated microglia are DAMs, microglia neurodegenerative phenotype (MGnD), and activated response microglia (ARMs).^6^, ^42^ The functional role of many upregulated DAM genes are also implicated in phagocytosis, chemotaxis, damage‐mediated cytokine release, while downregulated genes are typical of microglial homeostasis.^42^ During early, asymptomatic stages, increased microglia activation into DAM states can promote Aβ clearance and confer neuroprotection, which can shift to a neurodegenerative, proinflammatory state reminiscent of the MGnD phenotype as disease progresses further.^43^

Other myeloid cells contributing to neuroinflammation are non‐parenchymal macrophages. Under inflammatory and diseased conditions, bone‐marrow (BM)‐derived monocytes can infiltrate into the brain, differentiate into macrophages upon immune activation, and exhibit microglial‐like transcriptional and phenotype signatures.^36^, ^44^, ^45^, ^46^, ^47^ In *post mortem* AD tissue and pre‐clinical AD mouse models, circulating monocyte/macrophages have been identified infiltrating into brain parenchyma and localizing tightly amyloid plaques.^36^, ^46^, ^48^, ^49^ Notably, these BM‐derived macrophages appear to be more efficient than resident microglia in phagocytosis and clearance of Aβ within the brain parenchyma, while protecting synapses from loss.^49^, ^50^, ^51^ Conversely, disease stage may impact the phagocytic capacity of peripheral myeloid cells in clearing Aβ during the late‐stage disease.^52^ Glatiramer acetate was used to recruit immunomodulatory BM‐derived macrophages, but only modestly reduced Aβ burden in 5xFAD mice during the early development stage of pathology and increased Aβ levels during later stages of neuropathology.^53^ These results highlight the important, but nuanced role of infiltrating BM‐derived monocyte/macrophages in regulating neuroinflammation and shaping AD neuropathology.

This study also builds upon an ongoing question in the field about the role of infectious microbes, its resulting inflammation, and its effects on the progression and severity of AD pathologies. This “antimicrobial protection hypothesis” illustrates Aβ as a highly conserved effector molecule with innate immune functions that entrap invading pathogens for neutralization and sequestering via clearance.^54^ However, studies using viral infection models have shown mixed effects. HSV‐1 DNA has been reported to colocalize with Aβ plaques in *post mortem* AD brains, yet while potentially seeding surface glycoproteins within Aβ oligomers and accelerate deposition in HSV‐1‐infected 5xFAD brains.^55^, ^56^ Meanwhile, *post mortem* AD brain tissue with concurrent HSV encephalitis demonstrate HSV‐infected neurons localized near Aβ plaques and neurofibrillary tangles, but were not directly associated with worsening AD pathology.^57^ Interestingly, AD transgenes did not significantly protect against HSV‐1 exposure in mice nor initiate Aβ aggregation following infection, but did result in extensive infiltration of peripheral leukocytes and increased phagocytic activity of reactive microglia.^58^, ^59^ In our study, the presence of established Aβ deposition did not lead to significant control of JHMV replication and clearance in infected 10‐month‐old 5xFAD brains. We also did not observe colocalization of viral RNA with dense‐core Aβ plaques in subiculum nor brain stem regions of JHMV‐infected 5xFAD mice. It will be important to inoculate virus in 5xFAD brains before aggregation of fibrillar Aβ begins at 3‐months of age to determining whether viral material may impact Aβ deposition at earlier disease stages. Conversely, it may also reveal whether Aβ peptides may contribute to enhanced viral clearance before the onset of disease.

While we observed infiltration of CD4^+^ and CD8^+^ T cells in JHMV‐infected 5xFAD brains, intravenous labeling of the brain vasculature was not utilized to confirm whether these T cell populations were intravascular or infiltrating into the parenchyma. Therefore, the few CD4^+^ and CD8^+^ T cells observed in uninfected WT and 5xFAD mice may be circulating through the brain, while a small proportion within infected brains may also be associated within the vasculature. However, we also observed increased demyelination within spinal cord sections and increased protein concentration of plasma neurofilament light chain, suggesting extensive axonal damage likely attributed to viral RNA persisting within oligodendrocytes and white matter tracts.^20^, ^21^ JHMV‐mediated demyelination involves CD4^+^ T‐cell secretion of IFN‐γ and CCL5 to recruit peripheral macrophages which strip and engulf myelin from axons, while CD8^+^ T cells induce cytolytic activity within oligodendrocytes and OPCs.^60^, ^61^, ^62^ Recent evidence has also implicated demyelination in the etiology of pre‐clinical AD, demonstrating that lack of myelin integrity exacerbates Aβ plaque deposition in both AD brains and in AD mouse models.^63^ Given the connection between myelin damage in promoting Aβ plaque formation, future experiments will be necessary to investigate the extent through which the compaction of dense‐core Aβ plaques is mediated by JHMV‐induced encephalitis or the demyelination that follows.

While this study reports a dampened DAM transcriptional response to amyloid pathologies following encephalitis, there are several limitations that warrant future investigations. Here, we utilize single‐cell spatial transcriptomic imaging as a powerful method of retaining both spatial and transcriptional information within unique cell types. However, the pre‐selected 1000‐plex mouse neuroscience probe panel limits the depth of exploration and unbiased investigation compared to whole genome approaches offered by traditional single‐cell and single‐nucleus techniques. Specifically, the commercially available panel offers probes against key gene transcripts involved in neurological function, yet it is limited in probes for inflammation and immunity pathways and yields only surface‐level analyses on immune cell types. For example, we are unable to meaningfully parse out tissue‐resident microglial cells from infiltrating monocyte‐derived macrophages in the subclustered myeloid population due to limited markers specific to either peripheral monocyte/macrophages or brain‐resident microglia. In the Microglia population, we found an increase in cell counts within microglia subclusters across JHMV‐infected WT and 5xFAD groups. While microglial proliferation following viral infection has been reported, another possibility may be a result of clustering both microglia and peripheral monocyte/macrophages together due to limited probes and single‐cell resolution for this cell type. Interestingly, DGE analysis of each CNS cell type in the spatial transcriptomic dataset illustrates up‐regulation of antigen presentation genes *H2‐Aa* and *H2‐Ab1*, even among cells that are not typically associated with antigen presentation. These are likely due to segmentation overlaps with antigen‐presenting cells such as microglia and CNS‐associated macrophages, highlighting some limitations in the cell segmentation process for identifying captured cells within the tissue sample.

Recently, paternal inheritance of 5xFAD transgenes demonstrated greater plaque burden compared to 5xFAD mice inheriting transgenes from maternal mice.^64^ The mice used in this study were obtained through the MMRRC at The Jackson Laboratory before the organization strictly distributed 5xFAD mice strictly with paternal transgene inheritance. Future studies must account for these differences to demonstrate that our results are consistent across maternal and paternal inheritance of 5xFAD transgenes. Furthermore, it will be necessary to carefully consider and interpret the impact on AD pathologies not observed in the 5xFAD transgenic model, which lacks AD‐related tauopathies. Previously, our lab has reported on the impact of JHMV encephalitis on the 3xTg‐AD transgenic mouse model, which combines the autosomal dominant Swedish mutation in human *APP* to reproduce amyloid pathology and two human *MAPT* mutations associated with frontotemporal dementia (FTD) to reproduce tauopathies.^65^ While JHMV infection did not impact Aβ pathology in 3xTg‐AD brains, the resulting encephalitis did appear to worsen tau pathology.^65^ Additionally, its reliance on transgenes to overexpress rare autosomal‐dominant mutations limits the clinical relevance of this approach. With the advent of better humanized mouse models that have the potential of recapitulating amyloidosis and tauopathy together, future investigations investigate both the acute and post‐acute impact of viral encephalitis on these mice may offer more relevant and comprehensive understanding of its interactions with AD pathologies and its severity. Defining these post‐infectious neuroimmune mechanisms and their interaction with AD pathogenesis may offer potential treatments for current patients and preventative interventions to improve public health outcomes and reducing AD risk in an aging population.

## AUTHOR CONTRIBUTIONS

Dominic Ibarra Javonillo, Kim N. Green, and Thomas E. Lane designed the study; Susana Furman, Lucas Le, Kellie Fernandez, Jazmyn Mulford, Vidushi Singla, and Roshni Jha assisted Dominic Ibarra Javonillo in performing experiments; Dominic Ibarra Javonillo collected and analyzed experimental data; Dominic Ibarra Javonillo performed bulk RNA sequencing analysis; Dominic Ibarra Javonillo performed spatial transcriptomics; Dominic Ibarra Javonillo analyzed spatial transcriptomic data; Kate Inman Tsourmas and Nellie E. Kwang provided technical advice and scripts for spatial transcriptomic analysis. Dominic Ibarra Javonillo wrote the main manuscript text and prepared tables. All authors reviewed the manuscript.

## CONFLICT OF INTEREST STATEMENT

The authors declare that they have no conflicts of interest. Author disclosures are available in the Supporting Information.

## Supporting information




**Supporting Information**: alz71637‐sup‐0001‐SuppMat.pdf

**Supporting Information**: alz71629‐sup‐0002‐ICMJE.pdf
