# Supplementary material for: Acute viral encephalitis impacts dense‐core amyloid plaque pathology and dysregulates myeloid responses to amyloid plaques
Source: Alzheimers Dement. 2026 Jun 26;22(7):e71637. doi: 10.1002/alz.71637 (PMC13309288; doi:10.1002/alz.71637)
Supplement: Supplementary file 1 — Supporting Information: alz71637‐sup‐0001‐SuppMat.pdf [file ALZ-22-e71637-s001.pdf]

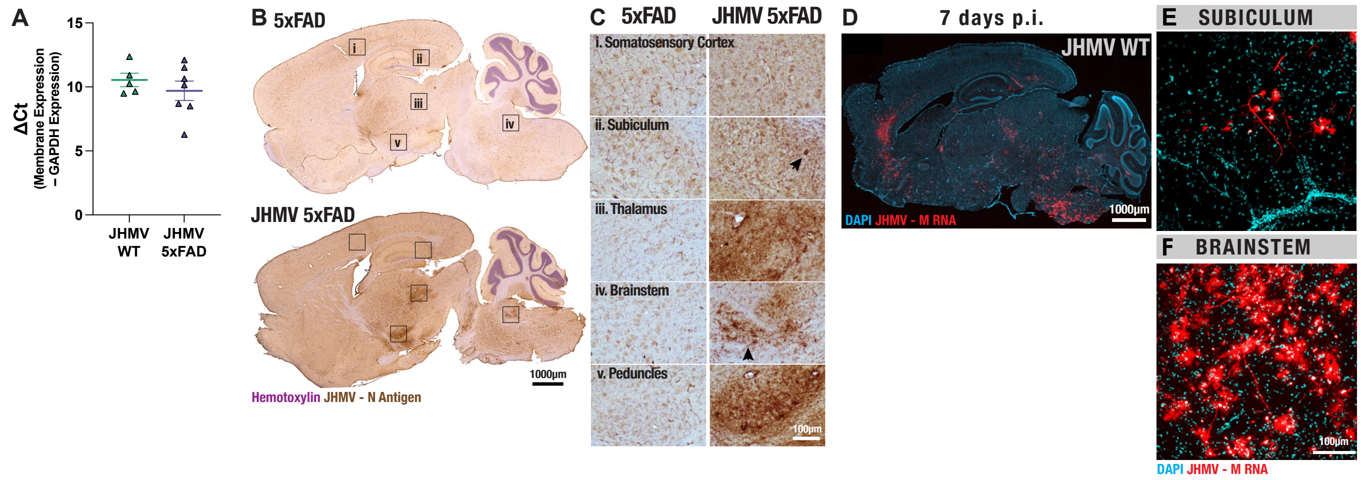
A
15
10
5
0
ΔCt
(Membrane Expression – GAPDH Expression)
JHMV WT
JHMV 5xFAD
B 5xFAD
i
ii
iii
iv
v
JHMV 5xFAD
1000µm
Hemotoxylin JHMV - N Antigen
C 5xFAD
JHMV 5xFAD
i. Somatosensory Cortex
ii. Subiculum
iii. Thalamus
iv. Brainstem
v. Peduncles
100µm
D 7 days p.i.
JHMV WT
1000µm
DAPI JHMV - M RNA
E SUBICULUM
F BRAINSTEM
100µm
DAPI JHMV - M RNA

Supplementary Figure 1. A) qPCR with extracted RNA from brain homogenates demonstrate similar transcript levels of viral membrane protein RNA between JHMV-infected WT and 5xFAD brains at 6-months of age at 10-14 dpi. **B)** Representative whole-brain scanned image of sagittal brain sections stained with H&E/DAB targeting JHMV nucleocapsid protein. **C)** Representative 10X brightfield images of different brain regions with or without presence of JHMV nucleocapsid protein (indicated with black arrowheads). **D)** Representative whole-brain scanned image of a sagittal brain section from a JHMV-infected WT mouse at 7 dpi depicting *fluorescent in situ hybridization* targeting JHMV viral RNA encoding membrane protein. Representative 20X confocal images of subiculum **(E)** and brainstem **(F)** visualizing the presence of JHMV RNA at 7dpi in an infected WT brain. n=7-10 per group n=4-10 per group. Data is presented as mean ± SEM. Unpaired t-tests was used to examine statistically significant differences between groups. Males are represented with closed symbols and females are represented with open symbols.

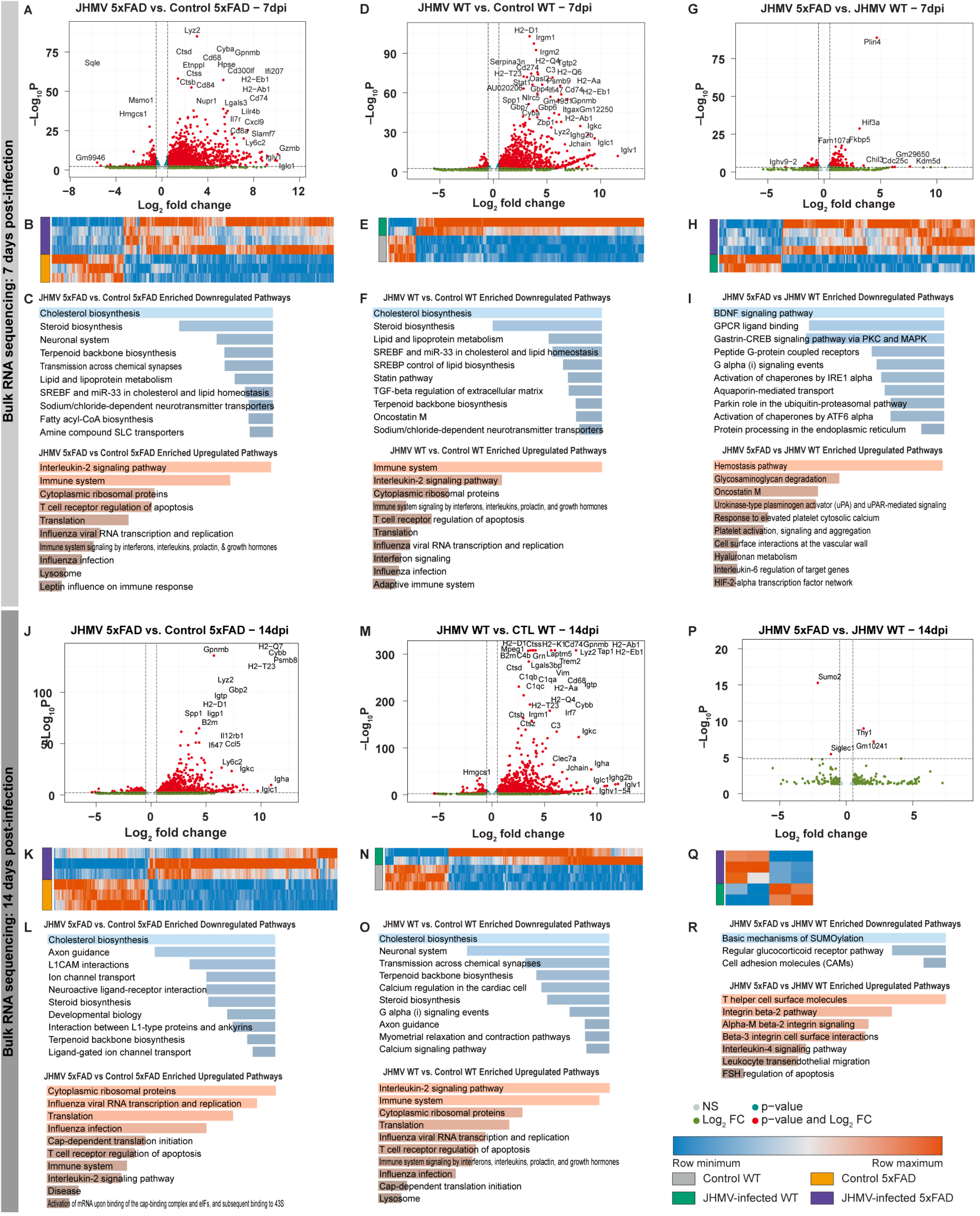

Bulk RNA sequencing: 7 days post-infection
A
JHMV 5xFAD vs. Control 5xFAD – 7dpi
Lyz2 Sqle Ctsd Cyba Gpnmb Cd68 Etnppl Hpse Cd300lf Ifi207 Ctss H2−Eb1 Ctsb Cd84 H2−Ab1 Msmo1 Nupr1 Lgals3 Cd74 Hmgcs1 Lilr4b Il7r Cxcl9 Cd8a Slamf7 Ly6c2 Gzmb Gm9946 Iglv1 Iglc1
−Log10P
Log2 fold change
B
C
JHMV 5xFAD vs. Control 5xFAD Enriched Downregulated Pathways
Cholesterol biosynthesis
Steroid biosynthesis
Neuronal system
Terpenoid backbone biosynthesis
Transmission across chemical synapses
Lipid and lipoprotein metabolism
SREBF and miR-33 in cholesterol and lipid homeostasis
Sodium/chloride-dependent neurotransmitter transporters
Fatty acyl-CoA biosynthesis
Amine compound SLC transporters
JHMV 5xFAD vs Control 5xFAD Enriched Upregulated Pathways
Interleukin-2 signaling pathway
Immune system
Cytoplasmic ribosomal proteins
T cell receptor regulation of apoptosis
Translation
Influenza viral RNA transcription and replication
Immune system signaling by interferons, interleukins, prolactin, & growth hormones
Influenza infection
Lysosome
Leptin influence on immune response
D
JHMV WT vs. Control WT – 7dpi
H2−D1 Irgm1 Irgm2 Serpina3n H2−Q4 Tgtp2 Cd274 H2−T23 C3 H2−Q6 Oasl2 Psmb9 H2−Aa Stat1 AU020206 Gbp4 Ifi47 Cd74 H2−Eb1 Spp1 Nlrc5 Gm4951 Gpnmb Gbp7 Gbp6 ItgaxGm12250 Cyba Zbp1 H2−Ab1 Igkc Lyz2 Ighg2b Jchain Iglc1 Iglv1
−Log10P
Log2 fold change
E
F
JHMV WT vs. Control WT Enriched Downregulated Pathways
Cholesterol biosynthesis
Steroid biosynthesis
Lipid and lipoprotein metabolism
SREBF and miR-33 in cholesterol and lipid homeostasis
SREBP control of lipid biosynthesis
Statin pathway
TGF-beta regulation of extracellular matrix
Terpenoid backbone biosynthesis
Oncostatin M
Sodium/chloride-dependent neurotransmitter transporters
JHMV WT vs. Control WT Enriched Upregulated Pathways
Immune system
Interleukin-2 signaling pathway
Cytoplasmic ribosomal proteins
Immune system signaling by interferons, interleukins, prolactin, and growth hormones
T cell receptor regulation of apoptosis
Translation
Influenza viral RNA transcription and replication
Interferon signaling
Influenza infection
Adaptive immune system
G
JHMV 5xFAD vs. JHMV WT – 7dpi
Plin4 Hif3a Fam107a Fkbp5 Ighv9−2 Chil3 Gm29650 Cdc25c Kdm5d
−Log10P
Log2 fold change
H
I
JHMV 5xFAD vs JHMV WT Enriched Downregulated Pathways
BDNF signaling pathway
GPCR ligand binding
Gastrin-CREB signaling pathway via PKC and MAPK
Peptide G-protein coupled receptors
G alpha (i) signaling events
Activation of chaperones by IRE1 alpha
Aquaporin-mediated transport
Parkin role in the ubiquitin-proteasomal pathway
Activation of chaperones by ATF6 alpha
Protein processing in the endoplasmic reticulum
JHMV 5xFAD vs JHMV WT Enriched Upregulated Pathways
Hemostasis pathway
Glycosaminoglycan degradation
Oncostatin M
Urokinase-type plasminogen activator (uPA) and uPAR-mediated signaling
Response to elevated platelet cytosolic calcium
Platelet activation, signaling and aggregation
Cell surface interactions at the vascular wall
Hyaluronan metabolism
Interleukin-6 regulation of target genes
HIF-2-alpha transcription factor network
Bulk RNA sequencing: 14 days post-infection
J
JHMV 5xFAD vs. Control 5xFAD – 14dpi
Gpnmb H2−Q7 Cybb Psmb8 H2−T23 Lyz2 Gbp2 Igtp H2−D1 Spp1 Iigp1 B2m Il12rb1 Ifi47 Ccl5 Ly6c2 Igkc Igha Iglc1
−Log10P
Log2 fold change
K
L
JHMV 5xFAD vs. Control 5xFAD Enriched Downregulated Pathways
Cholesterol biosynthesis
Axon guidance
L1CAM interactions
Ion channel transport
Neuroactive ligand-receptor interaction
Steroid biosynthesis
Developmental biology
Interaction between L1-type proteins and ankyrins
Terpenoid backbone biosynthesis
Ligand-gated ion channel transport
JHMV 5xFAD vs Control 5xFAD Enriched Upregulated Pathways
Cytoplasmic ribosomal proteins
Influenza viral RNA transcription and replication
Translation
Influenza infection
Cap-dependent translation initiation
T cell receptor regulation of apoptosis
Immune system
Interleukin-2 signaling pathway
Disease
Activation of mRNA upon binding of the cap-binding complex and eIFs, and subsequent binding to 43S
M
JHMV WT vs. CTL WT – 14dpi
H2−D1 Ctss H2−K1 Cd74 Gpnmb H2−Ab1 Mpeg1 Lyz2 Tap1 H2−Eb1 B2m C4b Grn Laptm5 Trem2 Ctsd Lgals3bp Vim C1qb C1qa Cd68 C1qc H2−Aa Igtp H2−Q4 H2−T23 Cybb Ctsb Irgm1 Irf7 Ctsz C3 Igkc Clec7a Hmgcs1 Jchain Igha Iglc1 Ighg2b Iglv1 Ighv1−54
−Log10P
Log2 fold change
N
O
JHMV WT vs. Control WT Enriched Downregulated Pathways
Cholesterol biosynthesis
Neuronal system
Transmission across chemical synapses
Terpenoid backbone biosynthesis
Calcium regulation in the cardiac cell
Steroid biosynthesis
G alpha (i) signaling events
Axon guidance
Myometrial relaxation and contraction pathways
Calcium signaling pathway
JHMV WT vs. Control WT Enriched Upregulated Pathways
Interleukin-2 signaling pathway
Immune system
Cytoplasmic ribosomal proteins
Translation
Influenza viral RNA transcription and replication
T cell receptor regulation of apoptosis
Immune system signaling by interferons, interleukins, prolactin, and growth hormones
Influenza infection
Cap-dependent translation initiation
Lysosome
P
JHMV 5xFAD vs. JHMV WT – 14dpi
Sumo2 Thy1 Siglec1 Gm10241
−Log10P
Log2 fold change
Q
R
JHMV 5xFAD vs JHMV WT Enriched Downregulated Pathways
Basic mechanisms of SUMOylation
Regular glucocorticoid receptor pathway
Cell adhesion molecules (CAMs)
JHMV 5xFAD vs JHMV WT Enriched Upregulated Pathways
T helper cell surface molecules
Integrin beta-2 pathway
Alpha-M beta-2 integrin signaling
Beta-3 integrin cell surface interactions
Interleukin-4 signaling pathway
Leukocyte transendothelial migration
FSH regulation of apoptosis
NS
p-value
Log2 FC
p-value and Log2 FC
Row minimum
Row maximum
Control WT
Control 5xFAD
JHMV-infected WT
JHMV-infected 5xFAD

Supplementary Figure 2. Bulk RNA sequencing was performed on extracted RNA from brain homogenates of 6-month-old WT and 5xFAD mice inoculated with either JHMV or vehicle controls at 7 or 14 days p.i. **A)** Volcano plot displaying fold change of DEGs (log_2 scale) at 7 days p.i. compared between JHMV 5xFAD vs. Control 5xFAD groups. **B)** Heatmap analysis depicts DEGs compared between JHMV 5xFAD vs. Control 5xFAD at 7 days p.i. Downregulated genes (log_2FoldChange < 0.5) are in blue, while upregulated genes (log_2FoldChange > 0.5) are in orange. **C)** Gene ontology (GO) pathway analysis depicts enriched pathways of down-regulated DEGs (blue) or up-regulated pathways (orange) at 7 days p.i. **D, G)** Volcano plot displaying fold change of DEGs (log_2 scale) at 7 days p.i. compared across respective groups (JHMV WT vs. Control WT and JHMV 5xFAD vs. JHMV WT, respectively). **E, H)** Heatmap analysis depicts DEGs compared across respective groups at 7 days p.i. Downregulated genes (log_2FoldChange < 0.5) are in blue, while upregulated genes (log_2FoldChange > 0.5) are in orange. **F, I)** Enriched pathways of either downregulated (in blue) or upregulated (in orange) DEGs at 7 days p.i. through gene ontology pathway analysis across each respective group comparison. **J)** Volcano plot displaying fold change of DEGs (log_2 scale) at 14 days p.i. compared between JHMV 5xFAD vs. Control 5xFAD groups. **K)** Heatmap analysis depicts DEGs compared between JHMV 5xFAD vs. Control 5xFAD at 14 days p.i. Downregulated genes (log_2FoldChange < 0.5) are in blue, while upregulated genes (log_2FoldChange > 0.5) are in orange. **L)** Gene ontology (GO) pathway analysis depicts enriched pathways of down-regulated DEGs (blue) or up-regulated pathways (orange) at 14 days p.i. **M, P)** Volcano plot displaying fold change of DEGs (log_2 scale) at 14 days p.i. compared across respective groups (JHMV WT vs. Control WT and JHMV 5xFAD vs. JHMV WT, respectively). **N, Q)** Heatmap analysis depicts DEGs compared across respective groups at 14 days p.i. Downregulated genes (log_2FoldChange < 0.5) are in blue, while upregulated genes (log_2FoldChange > 0.5) are in orange. **O, R)** Enriched pathways of either downregulated (in blue) or upregulated (in orange) DEGs at 14 days p.i. through gene ontology pathway analysis across each respective group comparison.

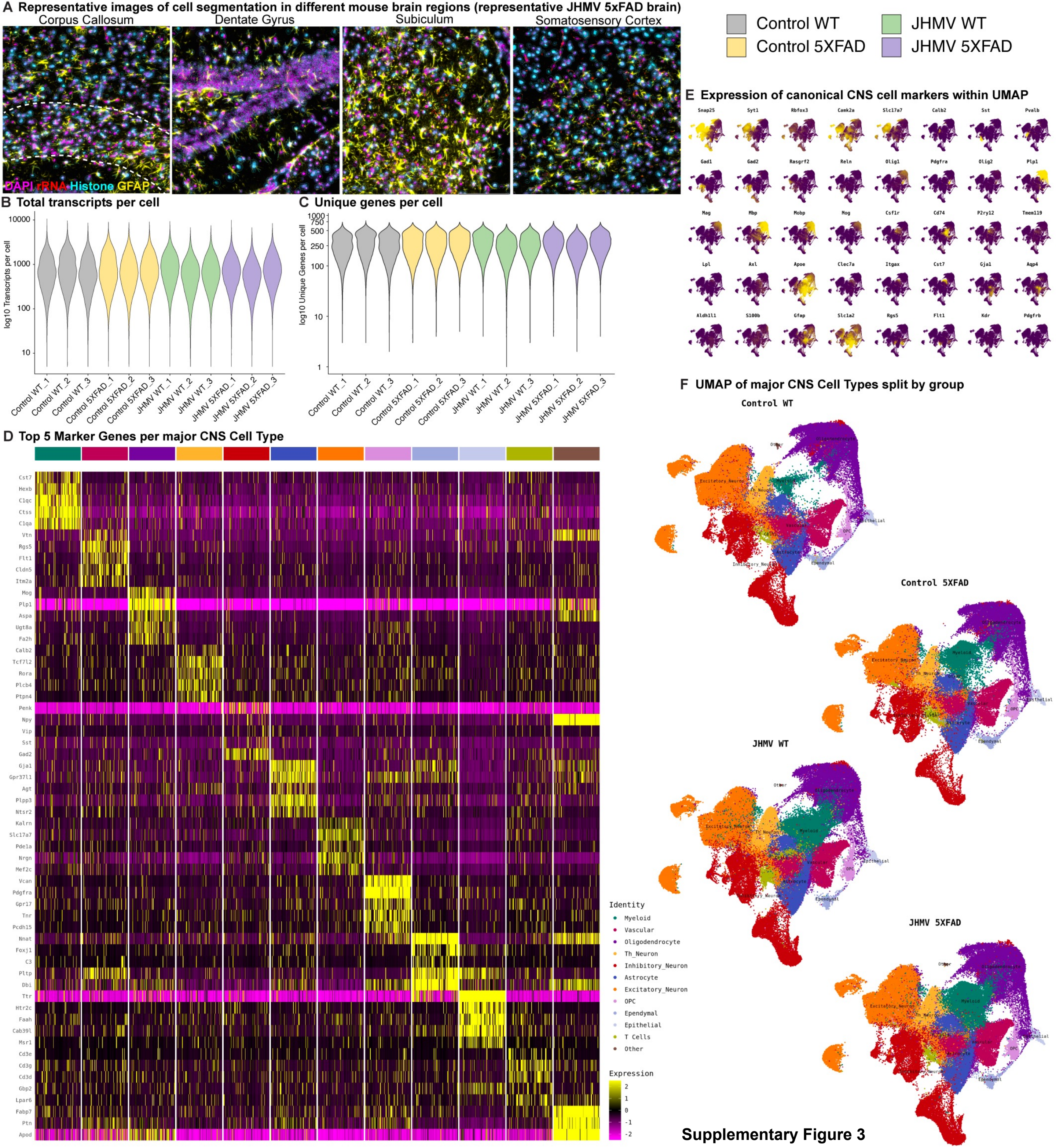

Supplementary Figure 3

Supplementary Figure 3. A) Representative images of cell segmentation within corpus callosum (dashed area), dentate gyrus, subiculum, and somatosensory cortex of a JHMV 5xFAD brain, respectively. **B)** Volcano plot depicting the total number of transcripts per cell across each sample. **C)** Volcano plot depicting the number of unique gene transcripts identified per cell across each sample. **D)** Heatmap depicting the top 5 expressed transcripts within each major CNS cell type to aid in the manual annotation of each cluster. **E)** Expression of canonical markers of common CNS cell types and their location in the UMAP. Included markers for neurons (*Snap25, Syt1*), excitatory neurons (*Rbfox3, Camk2a, Slc17a7*), inhibitory neurons (*Calb2, Sst, Pvalb, Gad1, Gad2*), other neurons (*Rasgrf2, Reln*), OPCs (*Olig1, Pdgfra*), oligodendrocytes (*Olig2, Plp1, Mag, Mbp, Mobp, Mog*), microglia (*Csf1r; Cd74, P2ry12, Tmem119*), disease-associated microglia (*Lpl, Axl, Apoe, Clec7a, Itgax, Cst7*), astrocytes (*Gjal1, Aqp4, Aldh1l1, S100b, Gfap, Slc1a2*), vascular cells (*Rgs5*), endothelial cells (*Flt1, Kdr*), and smooth muscle cells (*Pdgfrb*). **F)** UMAP of each major CNS cell type split by experimental group.

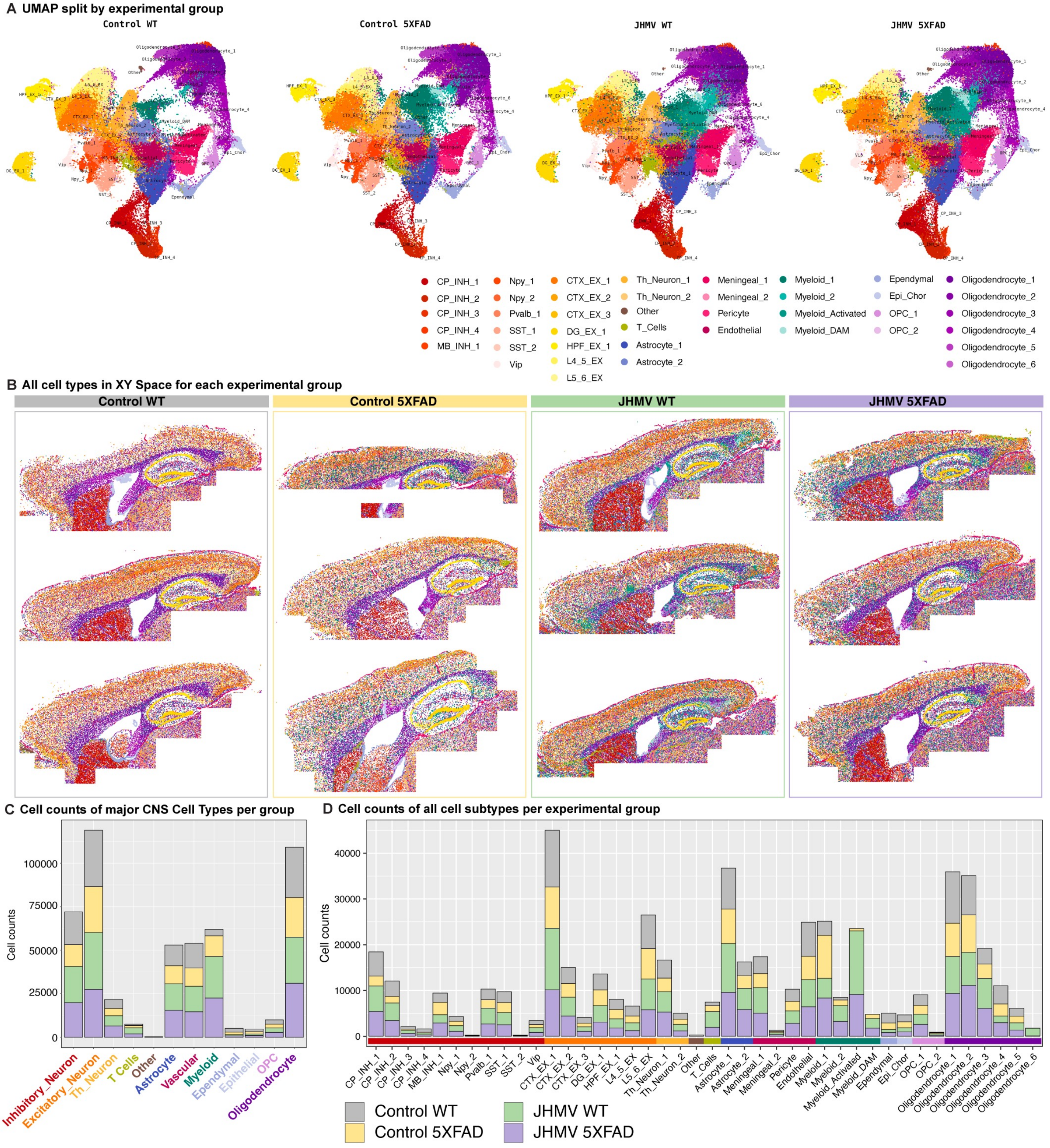
A UMAP split by experimental group
Control WT
Control 5XFAD
JHMV WT
JHMV 5XFAD
CP_INH_1
CP_INH_2
CP_INH_3
CP_INH_4
MB_INH_1
Npy_1
Npy_2
Pvalb_1
SST_1
SST_2
Vip
CTX_EX_1
CTX_EX_2
CTX_EX_3
DG_EX_1
HPF_EX_1
L4_5_EX
L5_6_EX
Th_Neuron_1
Th_Neuron_2
Other
T_Cells
Astrocyte_1
Astrocyte_2
Meningeal_1
Meningeal_2
Pericyte
Endothelial
Myeloid_1
Myeloid_2
Myeloid_Activated
Myeloid_DAM
Ependymal
Epi_Chor
OPC_1
OPC_2
Oligodendrocyte_1
Oligodendrocyte_2
Oligodendrocyte_3
Oligodendrocyte_4
Oligodendrocyte_5
Oligodendrocyte_6
B All cell types in XY Space for each experimental group
Control WT
Control 5XFAD
JHMV WT
JHMV 5XFAD
C Cell counts of major CNS Cell Types per group
Cell counts
100000
75000
50000
25000
0
Inhibitory_Neuron
Excitatory_Neuron
Th_Neuron
T Cells
Other
Astrocyte
Vascular
Myeloid
Ependymal
Epithelial
OPC
Oligodendrocyte
D Cell counts of all cell subtypes per experimental group
Cell counts
40000
30000
20000
10000
0
CP_INH_1
CP_INH_2
CP_INH_3
CP_INH_4
MB_INH_1
Npy_1
Npy_2
Pvalb_1
SST_1
SST_2
Vip
CTX_EX_1
CTX_EX_2
CTX_EX_3
DG_EX_1
HPF_EX_1
L4_5_EX
L5_6_EX
Th_Neuron_1
Th_Neuron_2
Other
T_Cells
Astrocyte_1
Astrocyte_2
Meningeal_1
Meningeal_2
Pericyte
Endothelial
Myeloid_1
Myeloid_2
Myeloid_Activated
Myeloid_DAM
Ependymal
Epi_Chor
OPC_1
OPC_2
Oligodendrocyte_1
Oligodendrocyte_2
Oligodendrocyte_3
Oligodendrocyte_4
Oligodendrocyte_5
Oligodendrocyte_6
Control WT
Control 5XFAD
JHMV WT
JHMV 5XFAD

Supplementary Figure 4. A) UMAP of each subtype within major CNS cell types, split by experimental group. **B)** All CNS subtypes overlaid in XY space within each brain sample in the spatial transcriptomic analysis. **C)** Plot depicting the number of cells associated with each major CNS cell type, split by experimental group. **D)** Plot depicting the number of cells within each CNS subtype, split by experimental group. Abbreviations include CP INH: caudate putamen inhibitory neurons, CTX EX: cortex excitatory neurons, DG EX: dentate gyrus excitatory neurons, Epi Chor: epithelial cells in choroid plexus, HPF EX: hippocampal formation excitatory neurons, L4 5 EX: layer 4/5 excitatory neurons, L5 6 EX: layer 5/6 excitatory neurons, MB INH: midbrain inhibitory neurons, Myeloid DAM: disease-associated myeloid cells, Npy: neuropeptide Y-expressing inhibitory neurons, OPC: oligodendrocyte precursor cells, Pvalb: parvalbumin-expressing inhibitory neurons, SST: somatostatin-expressing inhibitory neurons, Th Neuron: thalamus neurons, Vip: vasoactive intestinal polypeptide-expressing neurons.

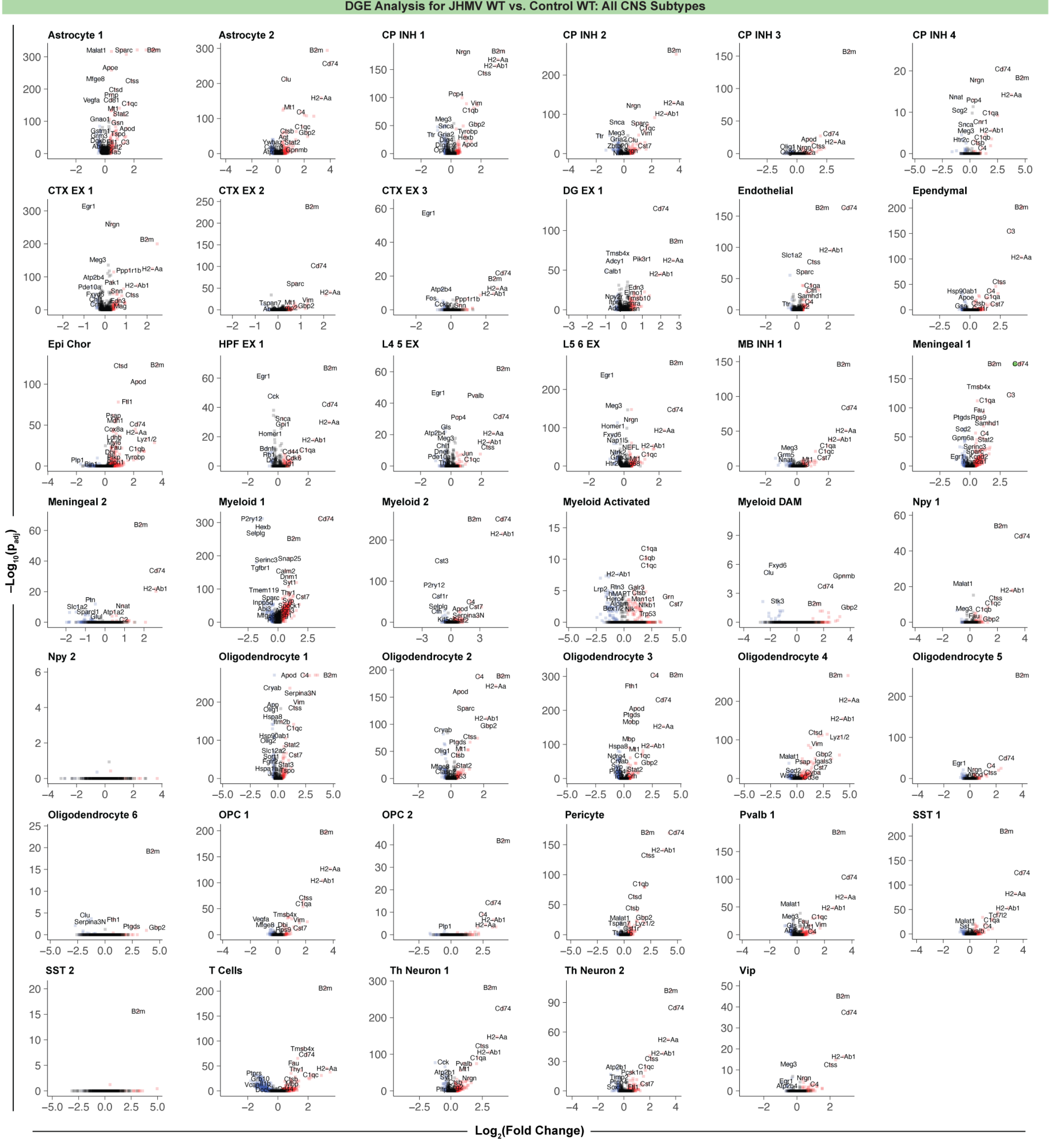
DGE Analysis for JHMV WT vs. Control WT: All CNS Subtypes
Astrocyte 1
Astrocyte 2
CP INH 1
CP INH 2
CP INH 3
CP INH 4
CTX EX 1
CTX EX 2
CTX EX 3
DG EX 1
Endothelial
Ependymal
Epi Chor
HPF EX 1
L4 5 EX
L5 6 EX
MB INH 1
Meningeal 1
Meningeal 2
Myeloid 1
Myeloid 2
Myeloid Activated
Myeloid DAM
Npy 1
Npy 2
Oligodendrocyte 1
Oligodendrocyte 2
Oligodendrocyte 3
Oligodendrocyte 4
Oligodendrocyte 5
Oligodendrocyte 6
OPC 1
OPC 2
Pericyte
Pvalb 1
SST 1
SST 2
T Cells
Th Neuron 1
Th Neuron 2
Vip
$-Log_{10}(p_{adj})$
Log_2(Fold Change)

Supplementary Figure 5. DGE Analysis of CNS subtypes for JHMV WT vs. Control WT at 12 days p.i. Volcano plots of DEGs within each CNS subtype type across JHMV-infected WT vs. control WT comparison at 12 days p.i. Abbreviations include: CP INH, caudate putamen inhibitory neurons; CTX EX, cortex excitatory neurons; DG EX, dentate gyrus excitatory neurons; Epi Chor, epithelial cells in choroid plexus; HPF EX, hippocampal formation excitatory neurons; L4 5 EX, layer 4/5 excitatory neurons; L5 6 EX, layer 5/6 excitatory neurons; MB INH, midbrain inhibitory neurons; Myeloid DAM, disease-associated myeloid cells; Npy, neuropeptide Y-expressing inhibitory neurons; OPC, oligodendrocyte precursor cells; Pvalb, parvalbumin-expressing inhibitory neurons; SST, somatostatin-expressing inhibitory neurons; Th Neuron, thalamus neurons; Vip, vasoactive intestinal polypeptide-expressing neurons.

Supplementary Figure 6

DGE Analysis for JHMV WT vs. Control WT

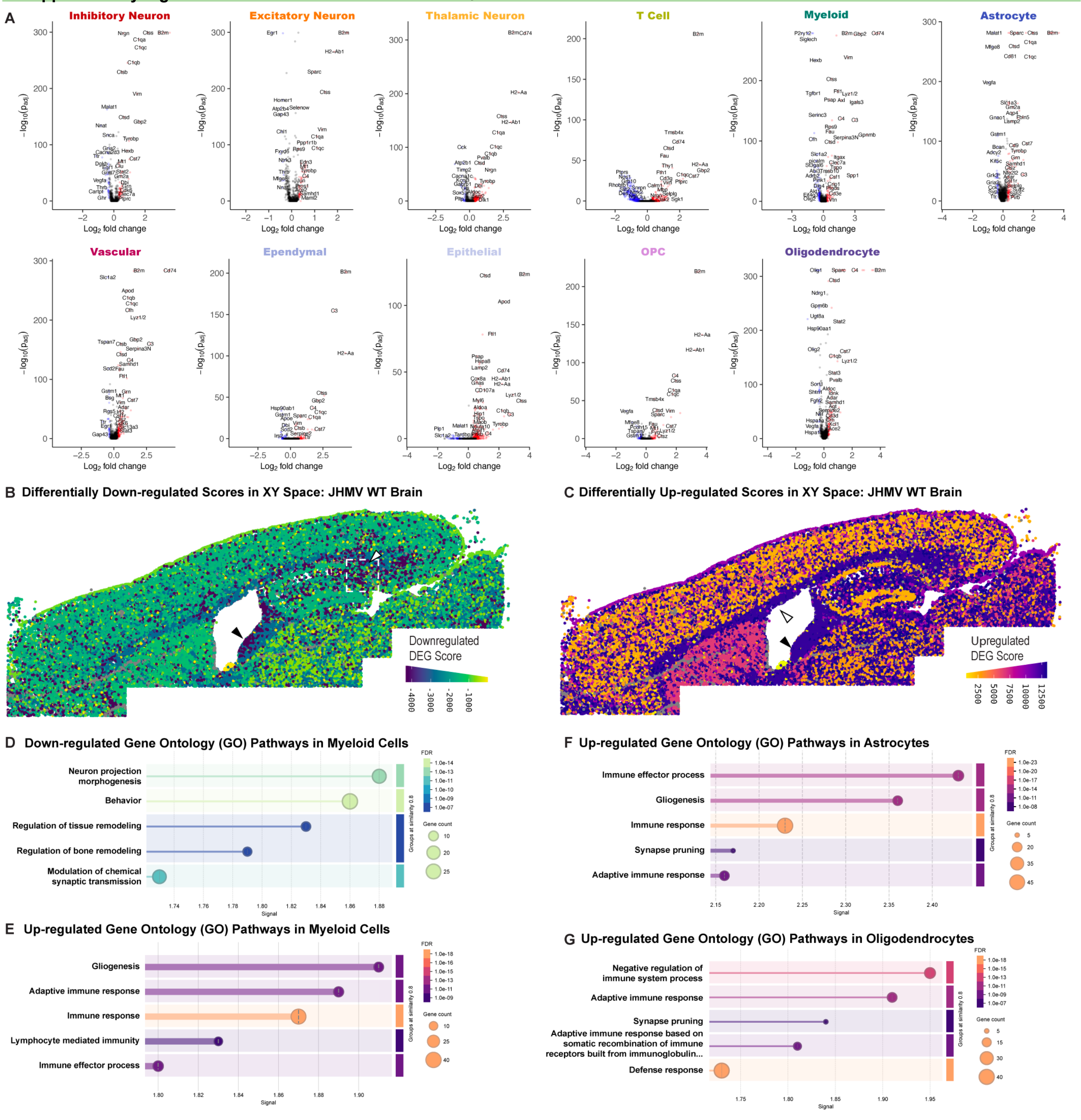

Supplementary Figure 6. DGE Analysis of each cell type for JHMV WT vs. Control WT comparison. **A)** Volcano plots of DEGs within each major CNS cell type across JHMV-infected 5xFAD vs. control 5xFAD. **B)** Differential downregulation (DD) and **C)** differential upregulation (DU) scores for JHMV-infected 5xFAD vs. control 5xFAD in each cluster plotted in XY space using a representative JHMV-infected 5xFAD brain. Arrowheads indicate major white matter tracts enriched in dysregulated cell types, specifically the fornix (black) and corpus callosum (white). **D)** GO Pathways enriched in down-regulated DEGs in myeloid cells. GO Pathways enriched in up-regulated DEGs in myeloid cells **(E),** astrocytes **(F)**, and oligodendrocytes **(G)**.

Supplementary Figure 7

DGE Analysis for Control 5XFAD vs. Control WT: All CNS Subtypes

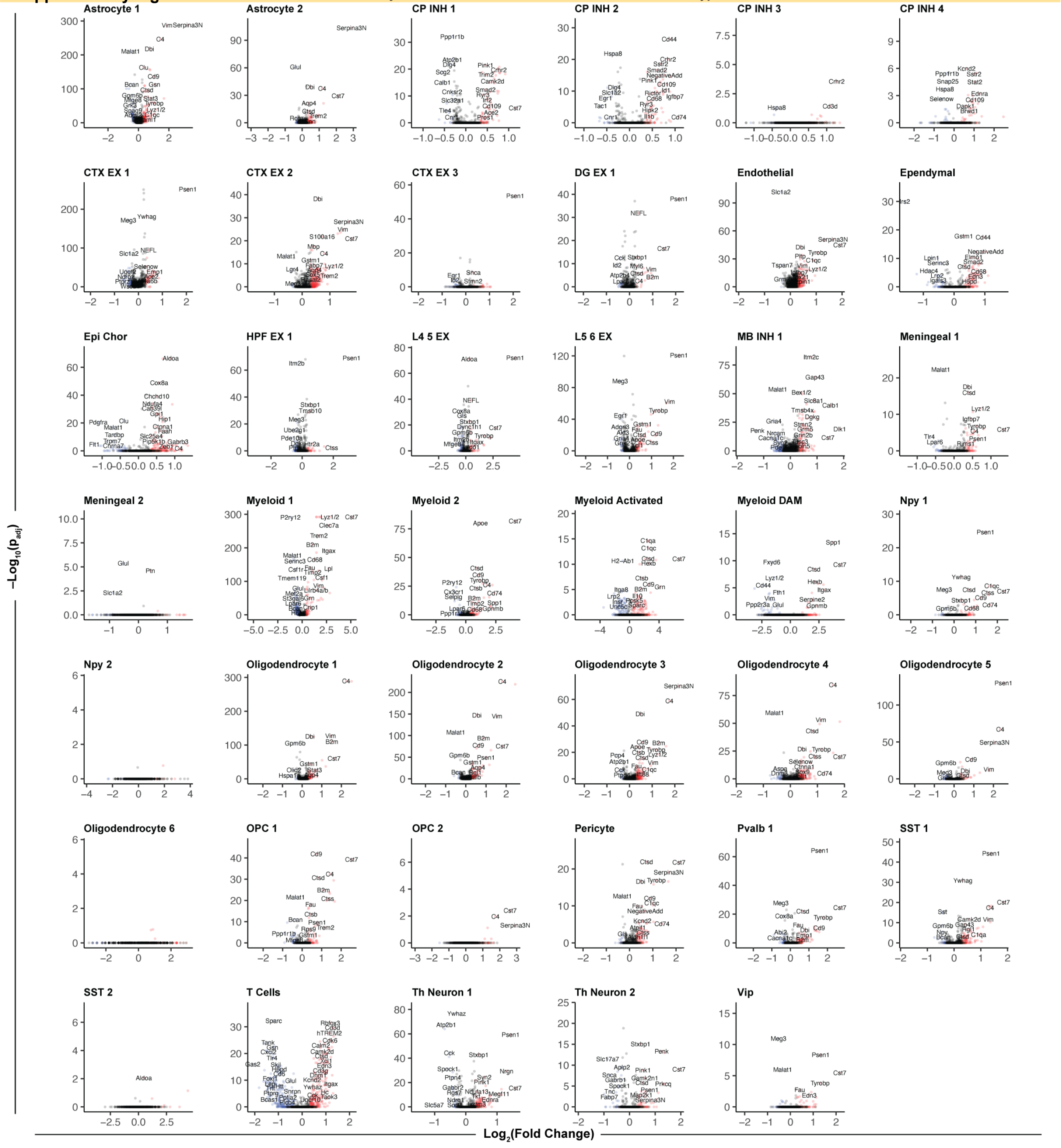

Supplementary Figure 7. DGE Analysis of CNS subtypes for Control 5xFAD vs. Control WT at 12 days p.i. Volcano plots of DEGs within each CNS subtype type across Control 5xFAD vs. control WT comparison at 12 days p. i. Abbreviations include: CP INH, caudate putamen inhibitory neurons; CTX EX, cortex excitatory neurons; DG EX, dentate gyrus excitatory neurons; Epi Chor, epithelial cells in choroid plexus; HPF EX, hippocampal formation excitatory neurons; L4 5 EX, layer 4/5 excitatory neurons; L5 6 EX, layer 5/6 excitatory neurons; MB INH, midbrain inhibitory neurons; Myeloid DAM, disease-associated myeloid cells; Npy, neuropeptide Y-expressing inhibitory neurons; OPC, oligodendrocyte precursor cells; Pvalb, parvalbumin-expressing inhibitory neurons; SST, somatostatin-expressing inhibitory neurons; Th Neuron, thalamus neurons; Vip, vasoactive intestinal polypeptide-expressing neurons.

Supplementary Figure 8 — DGE Analysis for Control 5XFAD vs. Control WT

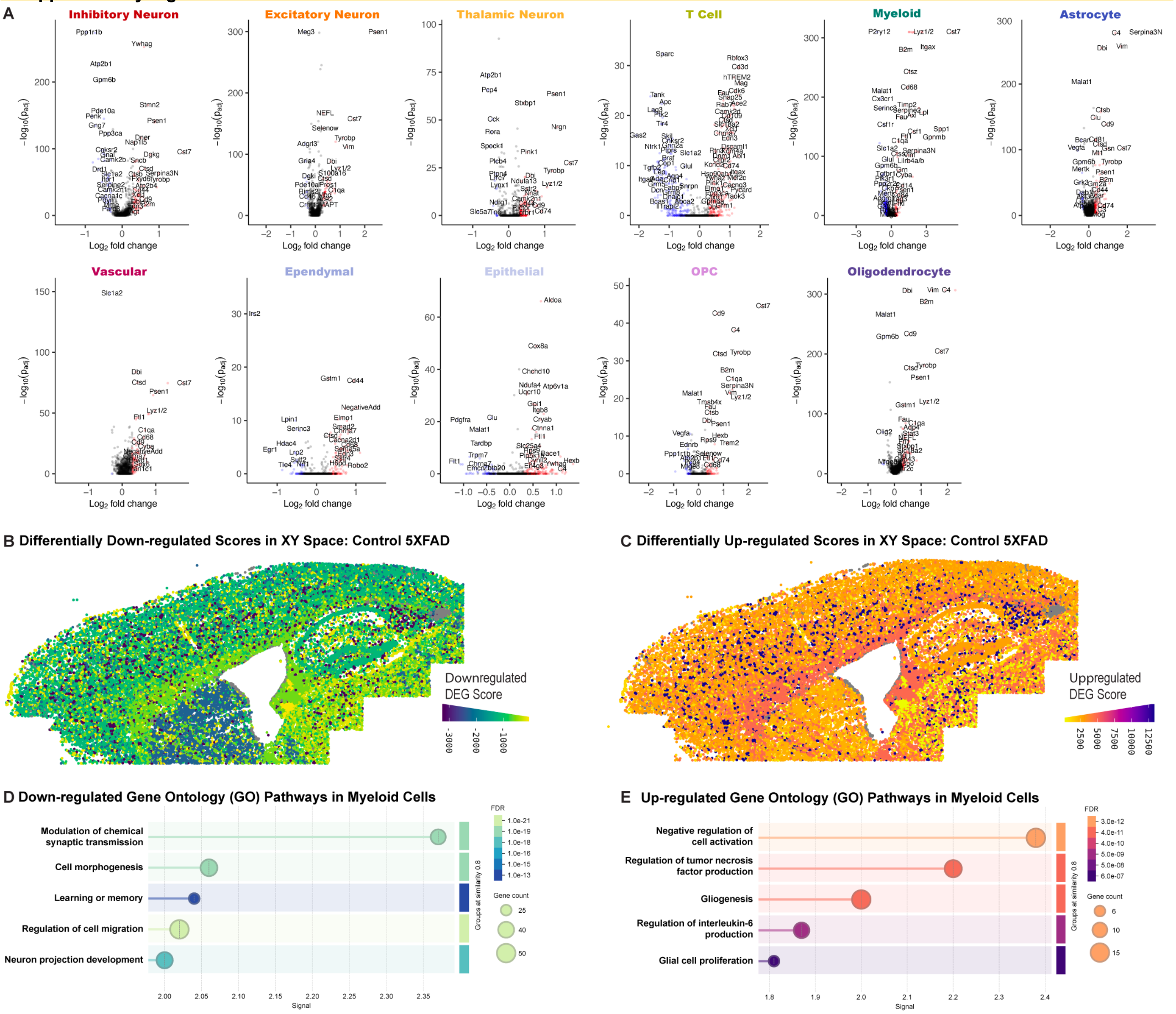

Supplementary Figure 8. DGE Analysis of each cell type for Control 5xFAD vs. Control WT comparison. A) Volcano plots of DEGs within each major CNS cell type across JHMV-infected 5xFAD vs. control 5xFAD. **B)** Differential downregulation (DD) and **C)** differential upregulation (DU) scores for JHMV-infected 5xFAD vs. control 5xFAD in each cluster plotted in XY space using a representative JHMV-infected 5xFAD brain. **D)** GO Pathways enriched in down-regulated DEGs in myeloid cells. **E)** GO Pathways enriched in up-regulated DEGs in myeloid cells.

Supplementary Figure 9

DGE Analysis for JHMV 5XFAD vs. Control 5XFAD: All CNS Subtypes

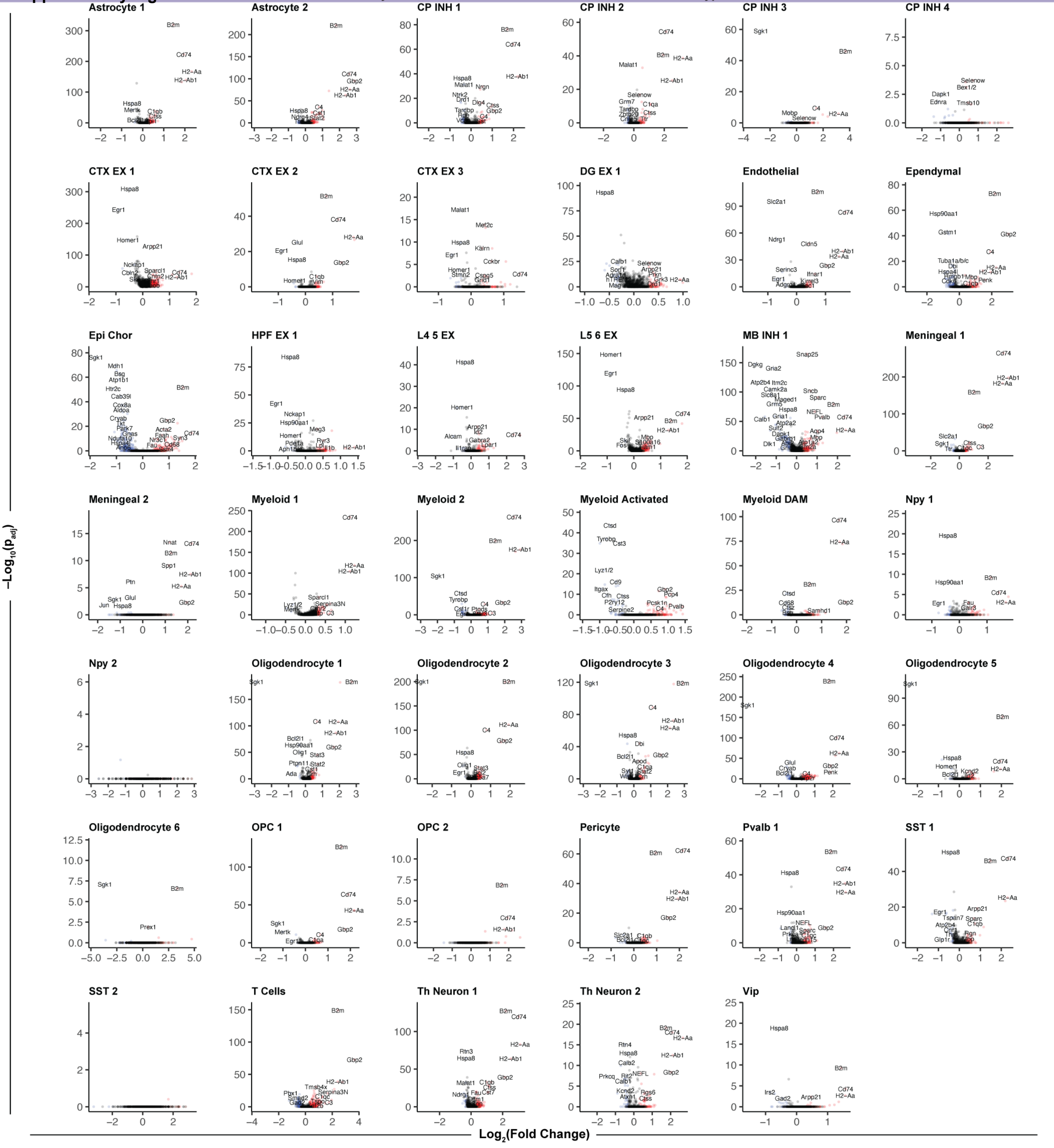

Supplementary Figure 9. DGE Analysis of CNS subtypes for JHMV 5xFAD vs. Control 5xFAD at 12 days p.i. Volcano plots of DEGs within each CNS subtype type across JHMV-infected 5xFAD vs. control 5xFAD comparison at 12 days p.i. Abbreviations include: CP INH, caudate putamen inhibitory neurons; CTX EX, cortex excitatory neurons; DG EX, dentate gyrus excitatory neurons; Epi Chor, epithelial cells in choroid plexus; HPF EX, hippocampal formation excitatory neurons; L4 5 EX, layer 4/5 excitatory neurons; L5 6 EX, layer 5/6 excitatory neurons; MB INH, midbrain inhibitory neurons; Myeloid DAM, disease-associated myeloid cells; Npy, neuropeptide Y-expressing inhibitory neurons; OPC, oligodendrocyte precursor cells; Pvalb, parvalbumin-expressing inhibitory neurons; SST, somatostatin-expressing inhibitory neurons; Th Neuron, thalamus neurons; Vip, vasoactive intestinal polypeptide-expressing neurons.

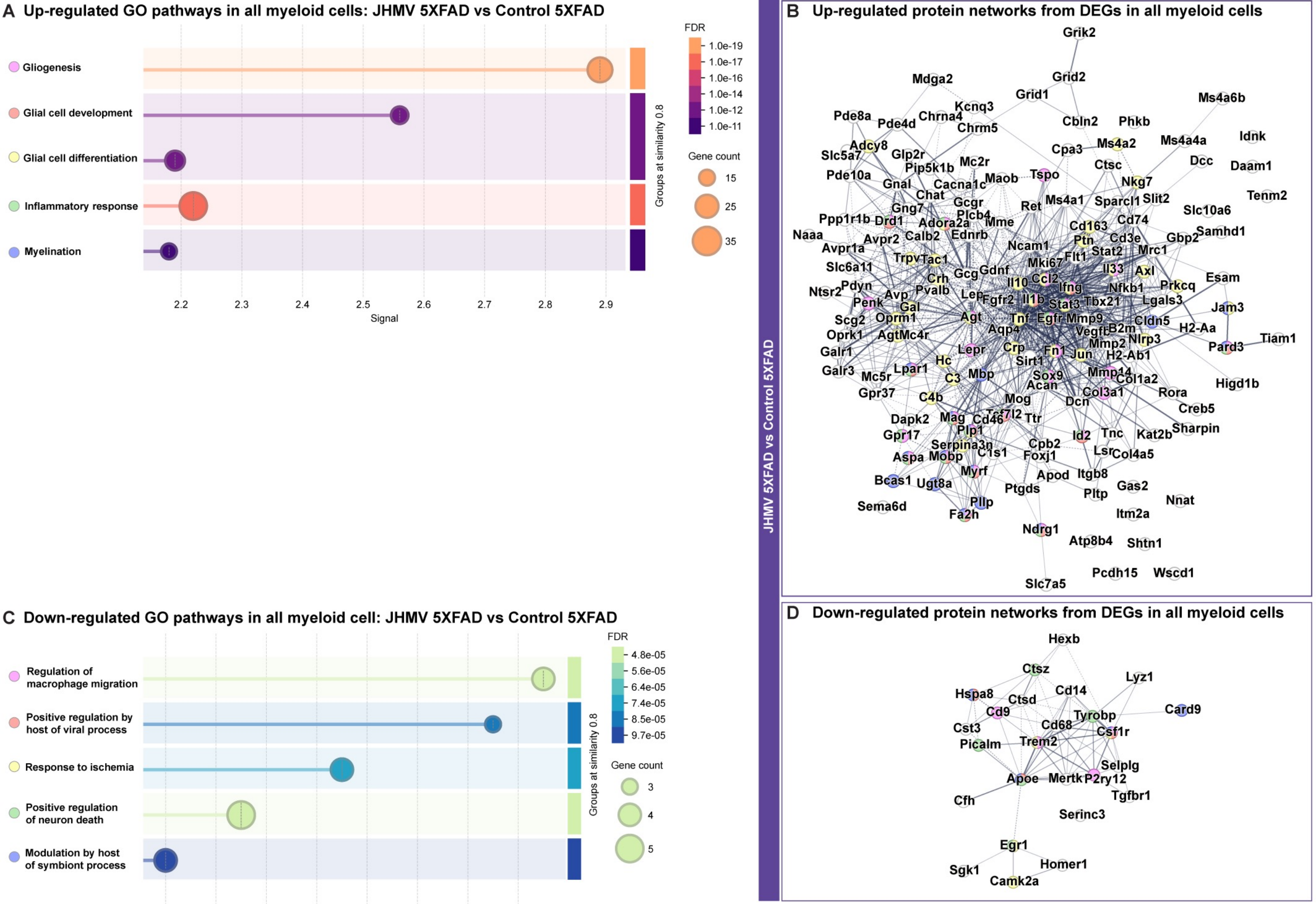

Supplementary Figure 10

Supplementary Figure 10. A) Pathway analysis by gene ontology for pathways enriched by significantly up-regulated DEGs within myeloid cells for JHMV 5xFAD vs. Control 5xFAD comparison. **B)** Protein-protein interactions (PPI) network of all significantly up-regulated DEGs with myeloid cells for the JHMV 5xFAD vs. Control 5xFAD comparison. **C)** Pathway analysis by gene ontology for pathways enriched by down-regulated DEGs within myeloid cells for JHMV 5xFAD vs. Control 5xFAD comparison. **D)** PPI network of all significantly down-regulated DEGs with myeloid cells for the JHMV 5xFAD vs. Control 5xFAD comparison. Nodes are colored according to its respective enriched pathway analysis. The thickness of each line connection between nodes indicates the degree of confidence in the prediction of the PPI network.

Myeloid clusters: Spatial transcriptomics analysis, Top 10 Expressed Genes, and Location in XY space

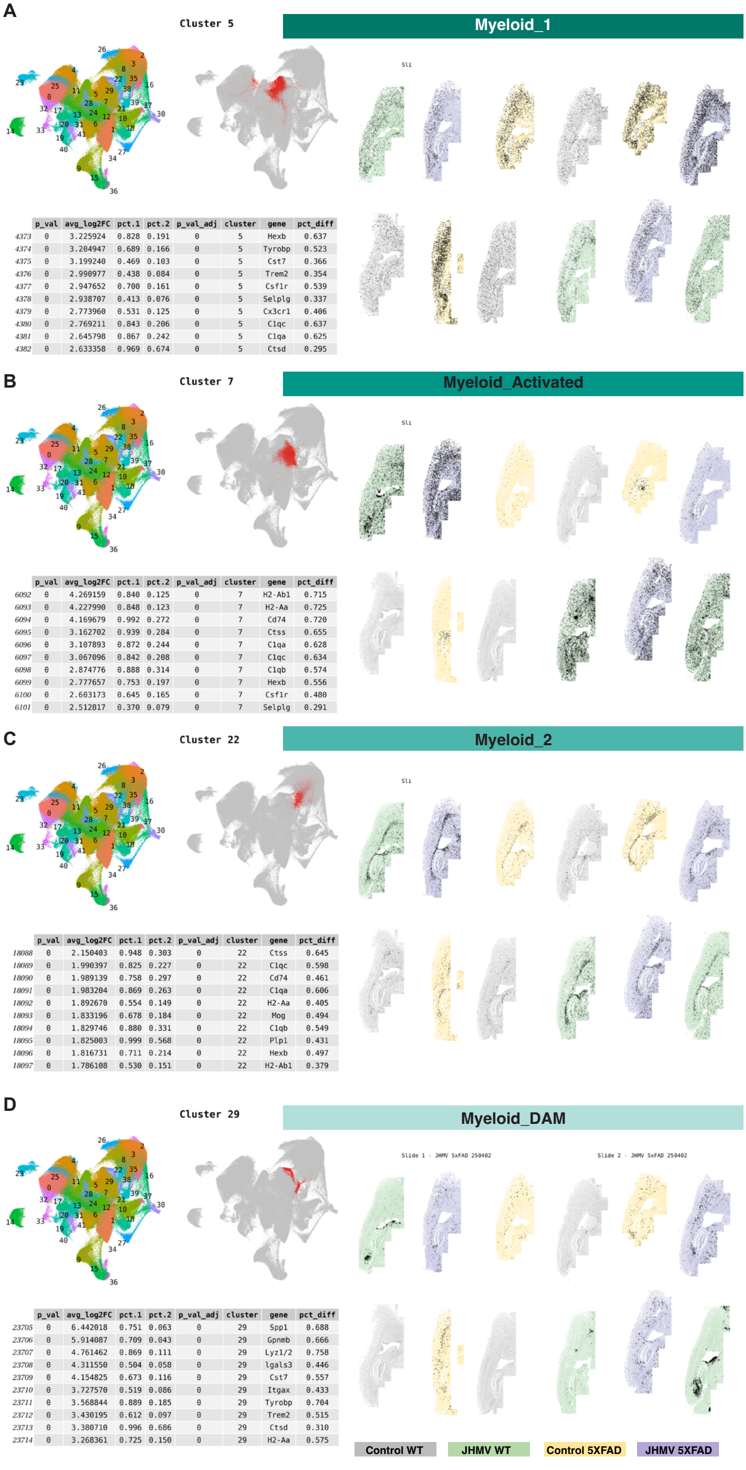

	p_val	avg_log2FC	pct.1	pct.2	p_val_adj	cluster	gene	pct_diff
4373	0	3.225924	0.828	0.191	0	5	Hexb	0.637
4374	0	3.204947	0.689	0.166	0	5	Tyrobp	0.523
4375	0	3.199240	0.469	0.103	0	5	Cst7	0.366
4376	0	2.990977	0.438	0.084	0	5	Trem2	0.354
4377	0	2.947652	0.700	0.161	0	5	Csf1r	0.539
4378	0	2.938707	0.413	0.076	0	5	Selplg	0.337
4379	0	2.773960	0.531	0.125	0	5	Cx3cr1	0.406
4380	0	2.769211	0.843	0.206	0	5	C1qc	0.637
4381	0	2.645798	0.867	0.242	0	5	C1qa	0.625
4382	0	2.633358	0.969	0.674	0	5	Ctsd	0.295

	p_val	avg_log2FC	pct.1	pct.2	p_val_adj	cluster	gene	pct_diff
6092	0	4.269159	0.840	0.125	0	7	H2-Ab1	0.715
6093	0	4.227990	0.848	0.123	0	7	H2-Aa	0.725
6094	0	4.169679	0.992	0.272	0	7	Cd74	0.720
6095	0	3.162702	0.939	0.284	0	7	Ctss	0.655
6096	0	3.107893	0.872	0.244	0	7	C1qa	0.628
6097	0	3.067096	0.842	0.208	0	7	C1qc	0.634
6098	0	2.874776	0.888	0.314	0	7	C1qb	0.574
6099	0	2.777657	0.753	0.197	0	7	Hexb	0.556
6100	0	2.603173	0.645	0.165	0	7	Csf1r	0.480
6101	0	2.512817	0.370	0.079	0	7	Selplg	0.291

	p_val	avg_log2FC	pct.1	pct.2	p_val_adj	cluster	gene	pct_diff
18088	0	2.150403	0.948	0.303	0	22	Ctss	0.645
18089	0	1.990397	0.825	0.227	0	22	C1qc	0.598
18090	0	1.989139	0.758	0.297	0	22	Cd74	0.461
18091	0	1.983204	0.869	0.263	0	22	C1qa	0.606
18092	0	1.892670	0.554	0.149	0	22	H2-Aa	0.405
18093	0	1.833196	0.678	0.184	0	22	Mog	0.494
18094	0	1.829746	0.880	0.331	0	22	C1qb	0.549
18095	0	1.825003	0.999	0.568	0	22	Plp1	0.431
18096	0	1.816731	0.711	0.214	0	22	Hexb	0.497
18097	0	1.786108	0.530	0.151	0	22	H2-Ab1	0.379

	p_val	avg_log2FC	pct.1	pct.2	p_val_adj	cluster	gene	pct_diff
23705	0	6.442018	0.751	0.063	0	29	Spp1	0.688
23706	0	5.914087	0.709	0.043	0	29	Gpnmb	0.666
23707	0	4.761462	0.869	0.111	0	29	Lyz1/2	0.758
23708	0	4.311550	0.504	0.058	0	29	lgals3	0.446
23709	0	4.154825	0.673	0.116	0	29	Cst7	0.557
23710	0	3.727570	0.519	0.086	0	29	Itgax	0.433
23711	0	3.568844	0.889	0.185	0	29	Tyrobp	0.704
23712	0	3.430195	0.612	0.097	0	29	Trem2	0.515
23713	0	3.380710	0.996	0.686	0	29	Ctsd	0.310
23714	0	3.268361	0.725	0.150	0	29	H2-Aa	0.575

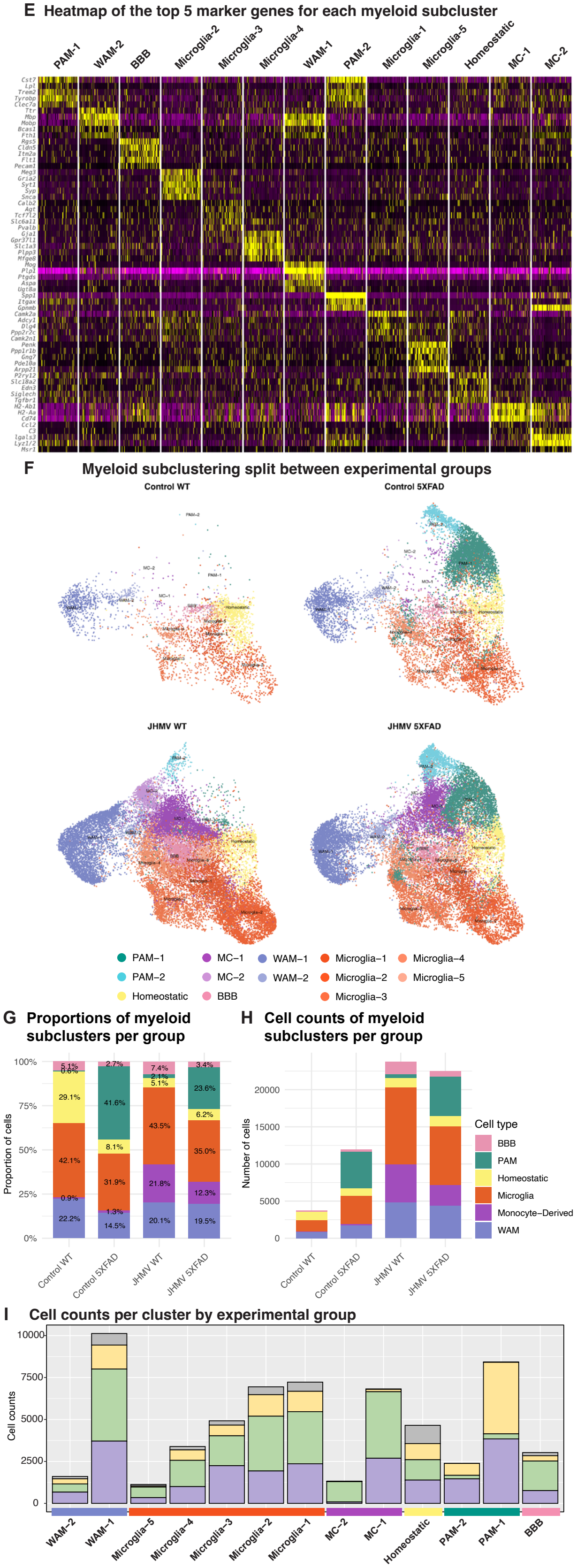

Supplementary Figure 11. Spatial transcriptomic analysis of the myeloid cluster. A) Cluster 5 location (highlighted in red) in the original UMAP annotated as Myeloid_1 based on the top 10 expressed genes and its distribution in XY space across all samples. **B)** Cluster 7 location in the original UMAP annotated as Myeloid_Activated based on the top 10 expressed genes and its distribution in XY space across all the samples. **C)** Cluster 22 location in the original UMAP annotated as Myeloid_2 based on the top 10 expressed genes and its distribution in XY space across all the samples. **D)** Cluster 29 location in the original UMAP annotated as Myeloid_DAM based on the top 10 expressed genes and its distribution in XY space across all the samples. **E)** Heatmap of the top 5 genes expressed in each myeloid subcluster. **F)** UMAP of myeloid subclusters split between each experimental group. **G)** Proportions of each myeloid cell type within each experimental group. **H)** Cell counts of each myeloid cell type within each experimental group. **I)** Plot depicting the cell counts of each myeloid subcluster split by experimental group. Abbreviations include: PAM, plaque-associated myeloid cells; MC, monocyte-derived cells; BBB, blood-brain-barrer-associated myeloid cells; WAM, white matter-associated myeloid cells.

Myeloid Subcluster Pseudo-bulk DGE Analysis: PAM & Monocyte-Derived Cells (MC)

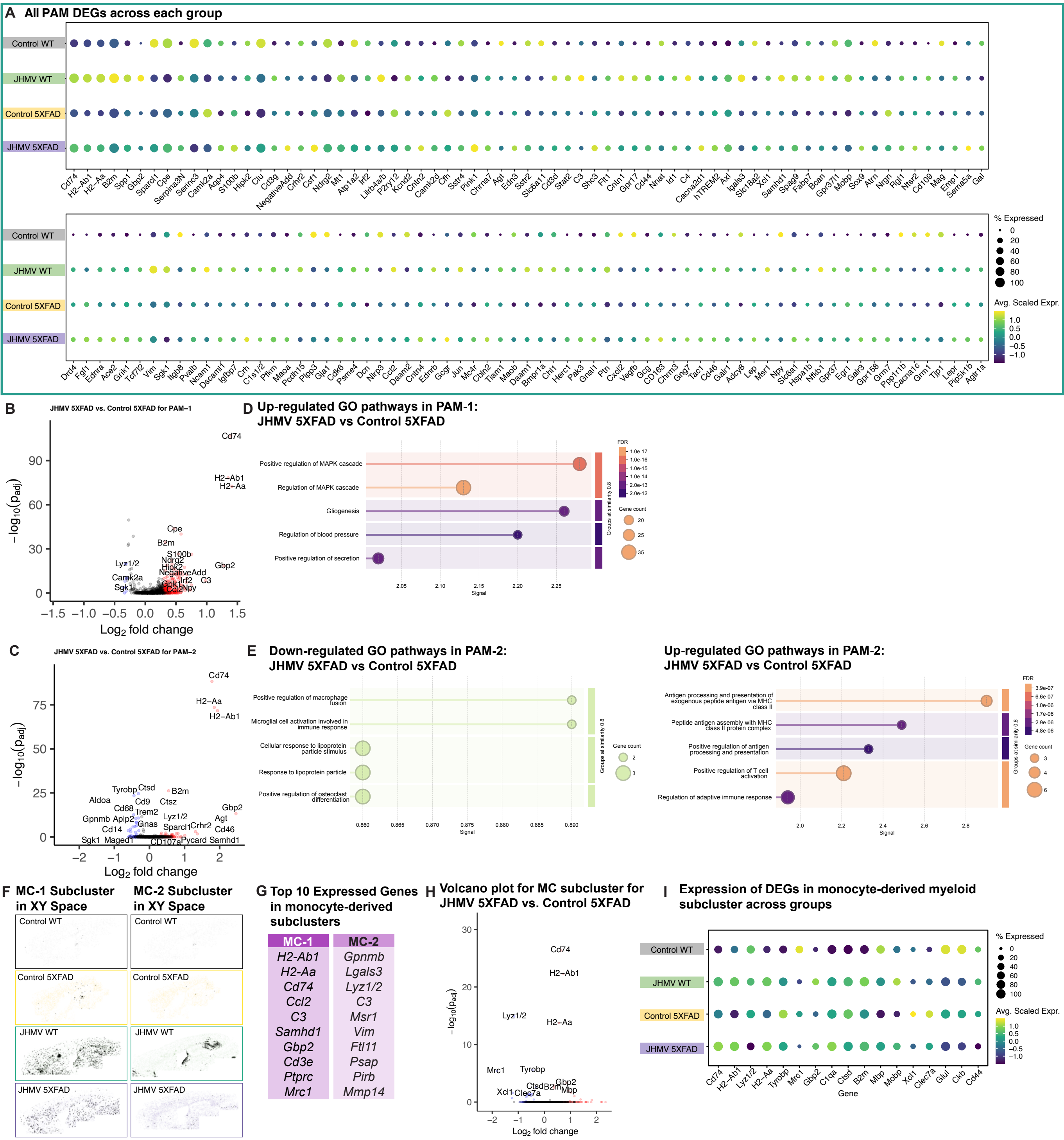

Supplementary Figure 12. Pseudo-bulk DGE analysis for Myeloid Subclusters PAM and Monocyte-derived cell. A) Pseudo-bulk sequencing analysis across all experimental groups depicting expression of DEGs within PAMs identified from the JHMV 5xFAD vs. Control 5xFAD comparison. **B-C)** Volcano plot depicting DEGs within PAM-1 (B) or PAM-2 (C) myeloid subcluster for the JHMV 5xFAD vs. Control 5xFAD comparison. **D)** Gene ontology (GO) pathway analysis of up-regulated DEGs in PAM-1 myeloid subcluster in the JHMV 5xFAD vs. Control 5xFAD comparison. **E)** GO pathway analysis for down-regulated (left) and up-regulated (right) DEGs in the PAM-2 myeloid subcluster in the JHMV 5xFAD vs. Control 5xFAD comparison. **F)** Monocyte-derived cells-1 (MC-1) and MC-2 subclusters in XY space on representative sections from each experimental group. **G)** List of the top 10 expressed genes in monocyte-derived cell subcluster MC-1 (left) and MC-2 (right). **H)** Volcano plot depicting DEGs in monocyte-derived subcluster for JHMV 5xFAD vs. Control 5xFAD comparison. **I)** Pseudo-bulk sequencing analysis across all experimental groups depicting expression of DEGs from the JHMV 5xFAD vs. Control 5xFAD comparison.

Supplementary Figure 13

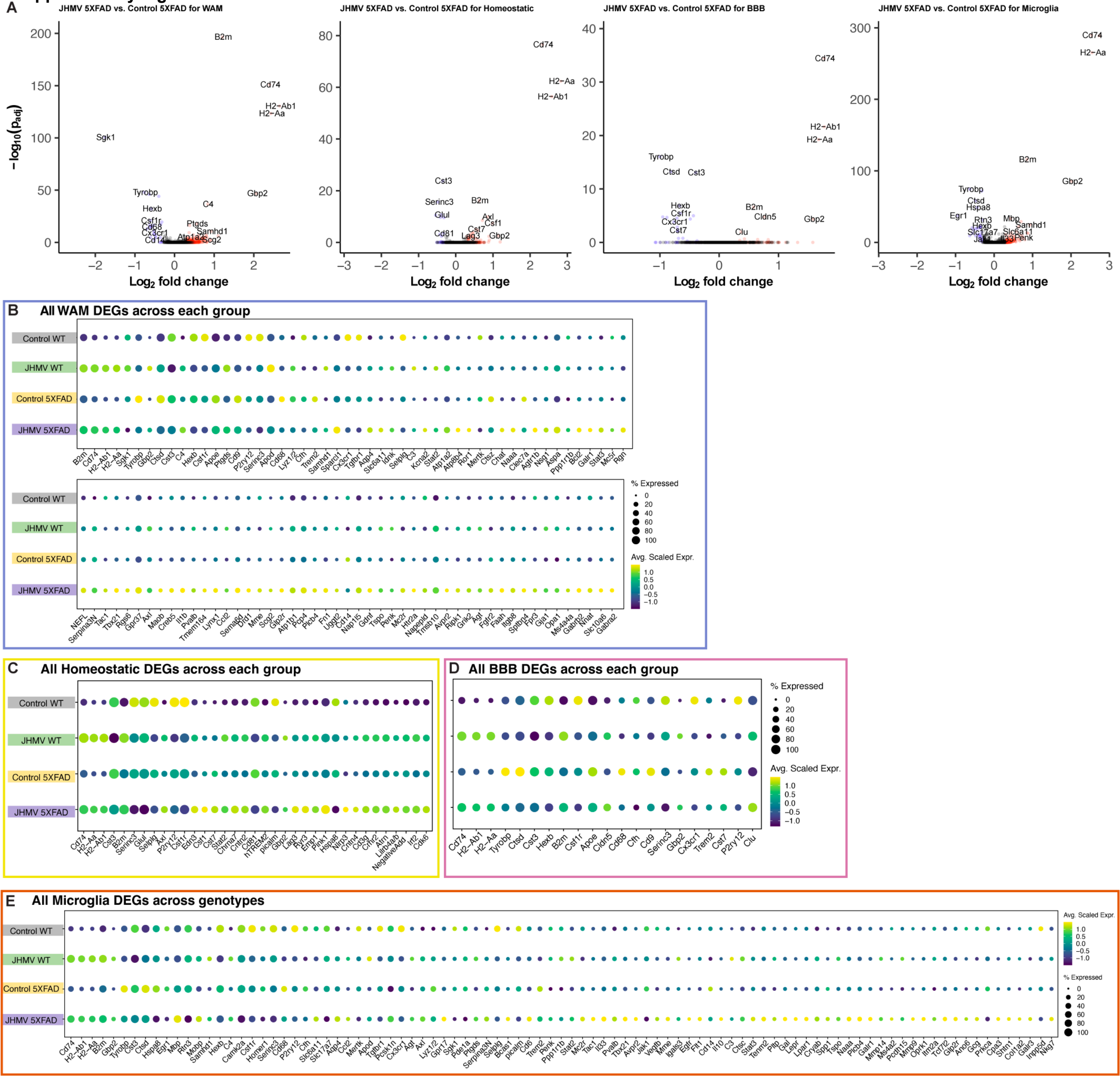

Supplementary Figure 13. A) Volcano plots displaying fold change of DEGs (log_2 scale) within WAM, homeostatic myeloid cells, BBB, and Microglia subclusters from the JHMV 5xFAD vs. Control 5xFAD comparisons. **B)** Pseudo-bulk sequencing analysis across all experimental groups depicting expression of DEGs within white-matter associated myeloid cells (WAMs) identified from the JHMV 5xFAD vs. Control 5xFAD comparison. **C)** Pseudo-bulk sequencing analysis across all experimental groups depicting expression of DEGs within homeostatic myeloid cells identified from the JHMV 5xFAD vs. Control 5xFAD comparison. **D)** Pseudo-bulk sequencing analysis across all experimental groups depicting expression of DEGs within myeloid cells associated with the blood-brain-barrier (BBB) identified from the JHMV 5xFAD vs. Control 5xFAD comparison. **E)** Pseudo-bulk sequencing analysis across all experimental groups depicting expression of DEGs within microglia identified from the JHMV 5xFAD vs. Control 5xFAD comparison.
